# Association of Acylcarnitine Species and Anthropometry Markers in a Population-Based Apparently Healthy Cohort

**DOI:** 10.3390/metabo16050315

**Published:** 2026-05-06

**Authors:** Ko Ko Maung, Rebecca Borreggine, Hector Gallart-Ayala, Julijana Ivanisevic, Pedro Marques-Vidal

**Affiliations:** 1 Department of Medicine, Internal Medicine, Lausanne University Hospital (CHUV), 1011 Lausanne, Switzerland; ko-ko.maung@chuv.ch; 2 Faculty of Biology and Medicine, University of Lausanne, 1015 Lausanne, Switzerland; 3 Metabolomics Platform, Faculty of Biology and Medicine, University of Lausanne, 1015 Lausanne, Switzerland; rebecca.borreggine@unil.ch (R.B.); hector.gallartayala@unil.ch (H.G.-A.); julijana.ivanisevic@unil.ch (J.I.)

**Keywords:** acylcarnitine, anthropometric markers, cross-sectional study, weight gain, prospective study

## Abstract

**Highlights:**

**What are the main findings?**

- Short-chain acylcarnitines showed strongest associations with multiple anthropometric markers.
- Higher baseline levels of short-chain and long-chain acylcarnitines were associated with future weight gain.

**What are the implications of the main findings?**

- Circulating acylcarnitine levels may capture early metabolic alterations linked to adiposity. These findings support future research into their potential role in preventive metabolic health assessment.

**Abstract:**

Background/Objectives: Acylcarnitine has been linked to adiposity, yet evidence in healthy adults is scarce. Hence, we aim to investigate associations of circulating acylcarnitine levels with traditional and newer anthropometric markers cross-sectionally, and with future weight change prospectively. Methods: We used data from CoLaus|PsyCoLaus cohort of apparently healthy adults in Lausanne, Switzerland. Anthropometry markers include body mass index, waist circumference, waist–hip ratio, conicity index, body roundness index, body shape index, leptin, adiponectin and grip strength. Results: Cross-sectionally, free carnitine, short-chain acylcarnitines (SCACs: C0, C3:0, C4:0 C5:0 and C5:0-OH), medium-chain acylcarnitines (MCACs: C6:0 and C8:1) and long-chain acylcarnitines (LCACs: C16:0) were positively associated with most anthropometric markers. After multivariate adjustment, only free carnitine, SCACs (C3:0 and C5:0), and MCAC C8:1 retained their positive associations with multiple markers. SCACs showed the strongest associations (−log10 *p*-values up to 91), followed by free carnitine and Deoxycarnitine. When stratified by sex, C8:1 showed consistent positive associations with anthropometric markers only in females. Prospectively, a higher baseline level of SCAC (C5:0-OH) was associated with ≥5 kg weight gain at both 5- and 10-year follow-ups, whereas higher baseline levels of MCAC (C8:1) and LCACs (C16:0 and C18:2) were associated with weight gain only at 10 years. Conclusions: SCAC showed most consistent associations with multiple anthropometric markers. Prospectively, specific ACs were associated with weight gain, suggesting that baseline AC levels may reflect early metabolic alterations linked to adiposity.

## 1. Introduction

One out of six people in Europe were either overweight or obese in 2022 according to the report by World Health Organization (WHO) [1]. In 2022, it is estimated that the prevalence of overweight was around 2.5 billion adults and around 390 million children and adolescents globally [2]. Mechanistically, excess adiposity is associated with dysregulation of mitochondrial β-oxidation and impairment of fatty acid metabolism, leading to alterations in the levels of specific fatty acid carrier metabolites known as acylcarnitine (AC) [3].

AC are esters formed by the conjugation of L-carnitine with acyl groups, which come from the metabolism of fatty acids or amino acid. They are essential intermediates in cellular energy metabolism, particularly recognized for their role in β-oxidation—a fundamental process for energy generation during fasting, physical exertion and metabolic stress [4]. AC are recognized especially for their role in energy production, metabolic regulation, and cognitive function, and are strongly associated with metabolic disorders and obesity [4]. Medium- and long-chain AC are elevated in the states of excess adiposity suggesting incomplete fatty oxidation and mitochondrial stress [5].

Traditional methods of assessing adiposity such as body mass index (BMI) and waist circumference have been widely used as an assessment of weight and overall weight-related risk and are shown to have strong predictive values for cardiometabolic risk [6]. Newer indices, conicity index (CI) and body roundness index, and a body shape index (ABSI) have been used recently for better assessment of adiposity and for better metabolic risk predictions [7]. Existing research shows a significant positive association of AC, particularly long-chained acylcarnitine (LCAC), with high BMI and waist circumference [8]. Furthermore, adipose tissues produce cellular signaling proteins called adipokines such as adiponectin and leptin. The adipokines are related to energy homeostasis, inflammation, and other metabolic processes [9]. The alterations in adipokines have also been shown to be associated with the disruption of fatty acid metabolism and mitochondrial function [10].

Existing evidence showed the relation of adiposity and the AC species in human. For instance, short-chain acylcarnitine (SCAC) such as Propionylcarnitine, Butyrylcarnitine, and medium-chain acylcarnitine (MCAC) such as Hexanoylcarnitine were associated with BMI, adiposity and leptin in Hispanic children [11]. The same AC species were also associated with BMI in pregnant women [12]. By contrast, LCAC showed strong associations with adiposity, and their levels were greater in obese and/or type 2 diabetic people [8]. However, the existing studies were conducted in populations such as children, pregnant women, or individuals with metabolic disorders, including obesity and type 2 diabetes. An exception is a study in a healthy adult European cohort which reported that free carnitine, SCACs and one LCAC (Palmitoylcarnitine) were associated with BMI.

To our knowledge, no study has ever been conducted on the association between comprehensive anthropometric makers and AC levels in a population-based, apparently healthy sample. Hence, the aim of the present study is to investigate the cross-sectional and prospective association between several anthropometric markers and circulating AC levels, for a better understanding of the metabolic implications of body composition.

## 2. Materials and Methods

### 2.1. Participants

The study used data from the CoLaus|PsyCoLaus study (www.colaus-psycolaus.ch), accessed on 1 May 2025, a prospective study conducted in the apparently healthy, community-dwelling population of Lausanne, Switzerland. Recruitment began in 2003 and ended in 2006 and included 6733 participants. The first follow-up (FU) was performed between 2009 and 2012 and included 5064 of the initial participants (75.2%). As the assessment of AC was conducted in the first follow-up, only data from this survey was used.

### 2.2. Acylcarnitine Measurements

#### 2.2.1. Sample and Calibration Curve Preparation

For absolute quantification of AC, samples were prepared by adding 250 μL of the diluted (1/500) stock IS mixture of AC in methanol to plasma (20 μL) as previously described [13]. Samples were then vortexed and centrifuged for 15 min at 4 °C and 2700× *g*. The resulting supernatant was transferred to LC-MS vials and injected into the Liquid Chromatography–High Resolution Mass Spectrometry Analysis (LC-HRMS) system. Ten-point calibration curves were generated following the same procedure as for the samples; in brief, 250 μL of 1/500 diluted IS mixture was added to each preprepared calibrator (10 μL of amino acid and 10 μL of AC calibrator, containing the increasing amount of each standard) and completed to 300 μL with 0.1% FA in water.

#### 2.2.2. Liquid Chromatography–High Resolution Mass Spectrometry Analysis

A Vanquish Horizon (Thermo Fisher Scientific, San Jose, CA, USA) ultrahigh-performance liquid chromatography (UHPLC) system coupled to Q-Exactive Focus interfaced with the heated electrospray ionization (HESI) source was used for the profiling of AC. The separation was carried out using a BEH Amide column (1.7 μm, 100 mm × 2.1 mm i.d.) (Waters, MA, USA). The mobile phase was composed of A = 20 mM ammonium format and 0.1% formic acid in water and B = 0.1% formic acid in acetonitrile (ACN). A gradient elution from 95% B (0–2 min) to 65% B (14 min) reaching 50% B at 16 min was applied and followed by 4 min post run for column re-equilibration. The flow rate was 400 μL/min, the column temperature was 25 °C, and the sample injection volume was 2 μL. HESI source conditions operating in positive mode were set as follows: sheath gas flow at 60, aux gas flow rate at 20, sweep gas flow rate at 2, spray voltage at +3 kV, capillary temperature at 300 °C, s-lens RF level at 60, and aux gas heater temperature at 300 °C. Full-scan HRMS (Manufacturer—Q-Exactive Focus from Thermo Fisher Scientific, San Jose, CA, USA) acquisition mode (*m*/*z* 50–750) was used with the following MS acquisition parameters: mass resolving power at 70,000 FWHM, 1 μscan, 1 × 10^6^ AGC, and 100 ms as maximum inject time.

The AC species analyzed in this study were classified into short-chain (SCAC), medium-chain (MCAC) and long-chain (LCAC), based on the chain length, reflecting distinct underlying metabolic pathways, as indicated in Supplementary Table S1.

#### 2.2.3. Measurement Accuracy and Precision

Briefly, the method accuracy was assessed by spiking at low and high concentration and ranged from 90 to 106%. Extraction recoveries range from 87 to 100%, and intra- and interday measurements from 1 to 10% and 1 to 15%, respectively. Overall, 20 authentic internal standards were used for quantification. Finally, measurement precision was also evaluated using internal quality control samples or pooled samples, which were analyzed periodically across 32 sample batches (or 32 96-well plates in total). The coefficient of variation for each acylcarnitine species is provided in Supplementary Table S7. Only species measured with high precision (CV < 20% over a period of 11 months) were kept for statistical analyses.

### 2.3. Anthropometry Markers

Anthropometry markers were selected to capture different dimensions of body composition and fat distribution. While BMI reflects general adiposity, other indices such as waist circumference, waist-to-hip ratio, and other related indices, provide additional information on central adiposity [7]. Therefore, 13 anthropometric indices were included in this analysis. Anthropometric measurements were conducted using a standard methodology, and the protocols were derived from NHANES III [14]

Body weight and height were measured with participants barefoot and in light indoor clothes. Body weight was measured in kilograms to the nearest 100 g using a Seca^®^ scale (Hamburg, Germany). Height was measured to the nearest 5 mm using a Seca^®^ (Hamburg, Germany) height gauge. Body mass index was computed and categorized as normal (<25 kg/m^2^) and overweight (25–29.9 kg/m^2^).

Waist circumference was measured mid-way between the lowest rib and the iliac crest using a non-stretchable tape and the average of two measurements was taken. Abdominal obesity was defined as a waist circumference >102 cm (men) or >88 cm (women).

The conicity index (CI) was calculated according to Valdez [15]. It is based upon the idea that people accumulate fat around the waist and, the shape of their bodies seems to change from that of a cylinder to that of a “double cone” (two cones with a common base). The CI is determined by the following formula.

CI = Waist/(0.109 × √((Body weight)/Height))

The CI was further categorized as normal if <1.25 and <1.18 for men and women, respectively, and as high if ≥1.25 and ≥1.18 for men and women, respectively, as proposed by Pitanga et al. [16].

Body roundness index (BRI) [7] was computed according to waist circumference and height.

$$BRI=364.2-365.5\times \sqrt{1-\left[\frac{{\left(\frac{Waist}{2\pi }\right)}^{2}}{{\left(0.5\times Height\right)}^{2}}\right]}$$

A Body Shape Index (ABSI) [17] was computed according to waist, BMI, and height. Since there are no clinical cutoff values for BRI or ABSI, high BRI and ABSI were defined as those within the highest quartile group (Q4).

ABSI=WaistBMI23×Height12

### 2.4. Bioelectrical Impedance Analysis Measurements

Bioelectrical impedance analyses (BIA) were performed as described by Bastardot et al. [18]. BIA was assessed using the Bodystat^®^ 1500 body mass analyzer, a single-frequency analyzer, (Bodystat Ltd., Isle of Man, UK) in the supine position after a 5 min rest. All metallic adornments were removed, and measurement was performed after a 5 min rest in the lying position. The electrodes were positioned on the right side of the body according to the manufacturer’s instructions. Results were obtained as percentage of body fat (%BF).

### 2.5. Clinical Markers of Adiposity

Leptin and Adiponectin levels were measured using a multiplexed particle-based flow cytometric cytokine assay [19]. Luminex Performance kits were purchased from Biotechne (Oxon, UK). The procedures closely followed the manufacturer’s instructions.

Assay ranges were 20–12,000 pg/mL for Leptin and 200–130,000 pg/mL for Adiponectin. Sample dilutions were 1:10 for Leptin and 1:2000 for Adiponectin. The analysis was conducted using a conventional flow cytometer (Guava EasyCyte 8HT, Millipore, Zug, Switzerland).

### 2.6. Grip Strength

Grip strength was used as proxy for muscle mass and was assessed using the Baseline^®^ Hydraulic Hand Dynamometer (Fabrication Enterprises Inc, Elmsford, NY, USA) with the subject seated, shoulders adducted and neutrally rotated, elbow flexed at 90°, forearm in neutral position and wrist between 0 and 30° of dorsiflexion. Three measurements were performed consecutively with the right hand, and the highest value (expressed in kg) was included in the analyses.

### 2.7. Other Covariates

Lifestyle data was self-reported. Smoking status was categorized as never, former and current. Presence of any type of diet (to reduce, low in lipids, other…) was categorized as yes or no. Alcohol consumption was categorized as present or absent. Physical activity was assessed by a questionnaire validated in the population of Geneva [20]. This questionnaire assesses the type and duration of 70 kinds of (non)professional activities and sports during the previous week. Sedentary status was defined as spending over 90% of the daily energy in activities below moderate- and high-intensity, defined as requiring at least 4 times the basal metabolic rate [21].

### 2.8. Exclusion Criteria

As the initial aim of the study was to establish reference values for healthy people, participants were eligible if their BMI was <30 kg/m^2^ and they had no diabetes. Eligible participants were excluded if: (1) they had no AC data; (2) they had no anthropometry data; and (3) the covariates needed for adjustment were lacking.

### 2.9. Statistical Analyses

Statistical analysis was conducted using Stata version 18.0 (Stata Corp, College Station, TX, USA). Summary statistics are reported as mean ± standard deviation or as median [interquartile range] for continuous variables and as number of participants (percentage) for categorical variables. Bivariate between-group comparisons were performed using the *t*-test or the Kruskal–Wallis test for continuous variables and the chi-square for categorical variables.

In cross-sectional analyses, bivariate associations between anthropometry markers and the AC species overall and stratified by sex were computed using Spearman correlation. The associations were further assessed using multivariable regression analysis adjusting for sex (except when stratified by sex), age (continuous), smoking status (never, former, current), presence of a diet (yes, no), and sedentary status (yes, no). Results were presented as standardized beta coefficients, as their interpretation is like correlation coefficients. *p*-values of the associations were adjusted for multiple comparisons using the False Discovery Rate method of Benjamini–Hochberg and considered as statistically significant if *p* < 0.001. For all other tests, statistical significance was considered for a two-sided test with *p* < 0.05.

In prospective analysis, bivariate associations between weight changes at 5- and 10-year follow-ups and the baseline acylcarnitine levels were computed using Wilcoxon rank-sum test. The associations were further assessed using multivariable ANOVA models adjusting for sex, age (continuous), smoking status (never, former, current), presence of a diet (yes, no), and sedentary status (yes, no). Results were presented as model-adjusted means of baseline acylcarnitine concentrations across three weight change categories (≥5 kg weight loss, stable weight, ≥5 kg weight gain) over 5-year (Follow-up 2-1) and 10-year (Follow-up 3-1) follow-ups.

For sensitivity analyses, early weight gainers, participants who gained over 5 kg during the first 5 years (n = 26), were excluded.

## 3. Results

### 3.1. Characteristics of the Participants

Out of the 5064 participants who attended the first follow-up of the CoLaus study during the period 2009–2012, 2482 participants were not eligible, and a further 349 were excluded due to a lack of data on either anthropometry or the covariates needed for adjustment, leaving 2233 participants for analysis. The reasons for exclusion are indicated in Supplementary Figure S1. Bivariate analysis between the excluded and included participants within eligible ones is summarized in Supplementary Table S2. Compared to the excluded participants, the included participants were younger, with fewer male participants, more current smokers, more alcohol drinkers, fewer on diet, were less sedentary and had a lower grip strength, BMI, WC and leptin levels.

The general characteristics and the distribution of anthropometric markers of the included participants, stratified by sex, are summarized in Table 1. Compared to males, females were older, more on a diet, and had a more sedentary lifestyle. Regarding anthropometric markers, females had lower BMI, hip and waist circumferences, conicity, BRI, ABSI and grip strength, and a lower frequency of overweight. In contrast, females had higher abdominal obesity, fat mass percentage, adiponectin and leptin levels. The levels of AC species according to sex are summarized in the Supplementary Table S3. Males had significantly higher levels of most AC species compared to females, except for adipoylcarnitine and hexadecenoylcarnitine, which were higher in women. No differences were found regarding acetylcarnitine, octanoylcarnitine, decanoylcarnitine and decenoylcarnitine.

### 3.2. Association Between Anthropometric Markers and Acylcarnitine Levels

The bivariate associations between anthropometric markers and AC species are shown in Table 2. Free carnitine showed positive associations with all anthropometric markers except with adiponectin. Most SCACs (deoxycarnitine, propionylcarnitine, butyrylcarnitine, glutarylcarnitine and hydroxyvalerylcarnitine), two MCACs (hexanoylcarnitine and octenoylcarnitine) and two LCACs (palmitoylcarnitine) showed positive associations with most anthropometric markers. Most AC species showed negative associations with fat mass (BIA) and adipokines, and positive associations with grip strength.

The associations between anthropometric markers and AC species were further assessed using multivariable regression analysis adjusted for potential confounders (Table 3). Free carnitine showed positive associations with all anthropometry markers except adiponectin. Certain AC species such as SCAC (propionylcarnitine and isovalerylcarnitine), and MCAC (octenoylcarnitine) retained their positive associations with most anthropometric markers. None of the positive associations between AC species with adiponectin and grip strength retained significance. SCAC (propionylcarnitine and isovalerylcarnitine) showed positive associations and MCAC (hydroxydodecanoylcarnitine) and LCAC (stearoylcarnitine) showed negative associations with leptin. Despite the modest effect sizes, the consistent direction of the associations across multiple anthropometric markers suggests an underlying association between circulating AC profiles and adiposity.

**Figure 1:**
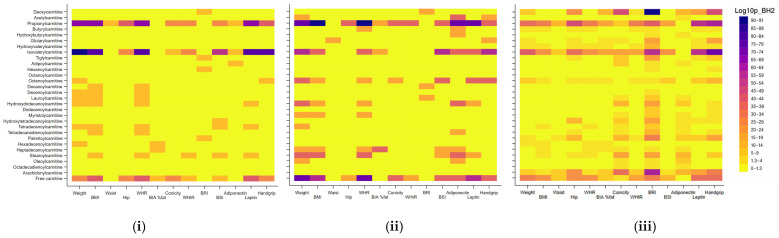
Significance of Beta correlation coefficients between anthropometric markers and acylcarnitines. Results are expressed as −log10(*p* values). (**i**) Male, (**ii**) female, (**iii**) overall.

When the strength of these associations was estimated using the *p*-values, isovalerylcarnitine and propionylcarnitine showed the strongest associations with anthropometric markers, with −log10 *p*-values of 91, followed by free carnitine and deoxycarnitine, the latter two being especially associated with grip strength (Figure 1).

Sensitivity analyses excluding early weight gainers, participants who gained at least 5 kg in the first follow-up, showed a similar pattern of associations compared to the primary analysis (Supplementary Table S6).

### 3.3. Association Between Anthropometric Markers and Acylcarnitine Levels, Stratified by Sex

The results of the multivariable analysis stratified by sex are presented in Supplementary Table S4 and Supplementary Figure S2. SCAC (propionylcarnitine and isovalerylcarnitine) showed consistent positive associations with most anthropometric markers for both sexes, and a negative association with adiponectin. Some species, such as octenoylcarnitine, showed consistent positive associations with most anthropometric markers in females but not in males.

When the strength of these associations was estimated using the *p*-values, propionylcarnitine and isovalerylcarnitine showed the strongest associations with most anthropometric markers with −log10 *p*-values of 90 for both sexes. Octenoylcarnitine showed stronger associations with anthropometric markers in females, whereas isovalerylcarnitine showed stronger associations in males than in females (Figure 1).

### 3.4. Association Between Changes in Weight and Acylcarnitine Levels

The bivariate associations between the baseline levels of AC levels and the weight changes over 5 years and 10 years are shown in Supplementary Table S5. The baseline means concentrations of MCAC (decanoylcarnitine, decenoylcarnitine, lauroylcarnitine and hydroxydodecanoylcarnitine) and LCAC (myristolycarnitine, hydroxytetradecanoylcarnitine, tetradecenoylcarnitine, hexadecenoylcarnitine, heptadecanoylcarnitine and stearoylcarnitine) were significantly lower in those who gained weight at least over 5 kg compared to the other groups, both at 5- and 10-year follow-ups.

The findings were further assessed in multivariable analysis adjusted for the potential confounders, evaluating the associations between baseline AC levels and subsequent weight changes over 5 and 10 years (Table 3). Compared to the participants who were weight-stable or lost weight, the baseline SCAC (hydroxyvalerylcarnitine) levels were higher among those who gained weight ≥ 5 kg at both 5 and 10 years. Baseline MCAC (octenoylcarnitine) levels were higher only among weight gainers at 10 years. At 10 years, the LCAC (palmitoylcarnitine and octadecadienoylcarnitine) levels were higher in those who gained ≥ 5 kg compared to weight-stable participants but lower than those who lost ≥ 5 kg. In addition, effect sizes were examined using a multivariable-adjusted model across weight change categories (≥5 kg loss or gain vs. stable weight) (Supplementary Table S10).

## 4. Discussion

Biologically, AC are important intermediates in cellular energy production, reflecting fatty acid β-oxidation in the mitochondrial matrix. Accumulation of AC species in the circulation has been interpreted as a marker of incomplete β-oxidation and increased flux of fatty acid. A prior study on AC in obesity and insulin resistance [8] suggested elevated circulating AC levels to be interpreted as mitochondrial overload and impaired regulation to downregulate under the conditions of increased BMI, waist circumference or insulin resistance. Several reports also showed an accumulation of AC species in metabolic disorders, including cardiovascular disease, heart failure and inherited metabolic disorders [3]. Importantly, our study extends these observations to an apparently healthy population-based sample devoid of obesity, diabetes and cardiovascular disease, aiming to investigate early metabolic alterations associated with adiposity.

The present study showed that SCAC and, to a lesser degree, MCAC were positively associated with most anthropometric markers, while only one LCAC species (stearoylcarnitine) showed an association in a sample of participants who were apparently healthy and not obese. The associations were comparable between sexes, except for octenoylcarnitine which showed associations with anthropometric markers in females but not in males. Prospectively, SCAC (hydroxyvalerylcarnitine) was associated with both 5- and 10-year weight changes, while MCAC (octenoylcarnitine) and LCAC (palmitoylcarnitine, hexadecenoylcarnitine and octadecadienoylcarnitine) were associated with 10-year weight changes. The elevated circulating SCAC may reflect their partial origin from the catabolic pathway of branched chain amino acid, which have been associated with adiposity and insulin resistance. In contrast, MCAC and LCAC are closely related to mitochondrial fatty oxidation and their accumulation may reflect impaired mitochondrial efficacy and altered energy metabolism [3].

### 4.1. Association Between Anthropometric Markers and Acylcarnitine Levels

In our study, SCAC (propionylcarnitine, butyrylcarnitine, isovalerylcarnitine and hydroxyvalerylcarnitine) and LCAC (palmitoylcarnitine) showed positive associations with most anthropometric markers. This agrees with a study conducted in healthy individuals in Europe, where SCAC (propionylcarnitine, butyrylcarnitine, and isovalerylcarnitine) and LCAC (palmitoylcarnitine) were associated with BMI [10]. However, in the studies conducted in obese and/or type 2 diabetes participants, LCAC and MCAC showed stronger associations with anthropometric markers than SCAC [8], whereas in our study the associations with SCAC were the strongest as assessed by the very low *p*-values. A likely explanation is that in patients with obesity or T2DM, excessive dietary fatty acid intake or insulin resistance leads to an overload of β-oxidation, resulting in increased levels of LCAC in the blood as a compensatory mechanism. Hence, the higher levels of SCAC found in this study among people with overweight suggest that these people are still able to adequately metabolize fatty acids, and the increased SCAC levels are a marker of an increased metabolic rate, possibly due to an increased muscle mass in people with overweight relative to people with normal weight. Eventually, MCAC and LCAC levels would progressively increase and overtake SCAC as the former two play a major role in proinflammatory role in case of fatty acid overload or insulin resistance [22].

### 4.2. Association Between Anthropometric Markers and Acylcarnitine Levels, Stratified by Sex

The findings are similar between sexes, with one exception of MCAC C8:1. However, the sex difference in the association could be either due to a larger sample size in females or to hormonal effects, as its levels increase considerably in post-menopausal women to values close to men (Supplementary Table S5). Our study also found significantly higher levels of AC species and higher anthropometric markers in the post-menopausal females, which agrees with a recent systematic review on adiposity in pre-and post-menopausal [23]. The increase in AC species in post-menopausal females could be related to a higher prevalence of insulin resistance in females after menopause [24], although the hypothesis is yet to be tested.

### 4.3. Association Between Changes in Weight and Acylcarnitine Levels

In our study, higher levels of MCAC and LCAC were associated with weight gain ≥ 5 kg over 10 years. Our findings agree with a large prospective study in Australia, which reported a positive association between MCAC and LCAC levels with both waist circumference and BMI [25]. Such an increase in AC metabolism may reflect the underlying mitochondrial dysfunction such as impaired β-oxidation, leading to fat accumulation [26]. In contrast, our findings showed that the baseline levels of LCAC were higher in participants who lost ≥ 5 kg compared with both weight-stable participants and weight gainers. Notably, a similar pattern was also reported in an interventional study of overweight participants with low-fat diet, where increased levels of MCAC and LCAC were associated with weight loss accompanied by rising free fatty acids and decreased visceral fats, consistent with fatty acid mobilization during weight loss [27]. Our findings may therefore capture a pre-existing high flux of fatty acid mobilization and oxidation at baseline that favors subsequent weight loss. Nevertheless, the findings should be interpreted cautiously since increased LCAC levels can also arise from incomplete β-oxidation.

### 4.4. Future Research Direction

The current study provides observational evidence from both cross-sectional and prospective associations between anthropometric markers and AC species. The findings support the potential role of AC as an early biomarker of future weight change. Nevertheless, future studies should validate the findings in independent cohorts across diverse populations to assess generalizability. As AC concentrations were measured only at baseline in the present study, future research could examine repeated longitudinal assessments of AC profiles to capture AC trajectories over time. Such studies could also clarify whether AC act as an early biomarker, mediator or potential contributor to subsequent weight change.

### 4.5. Strengths and Limitations

Most studies regarding the associations between anthropometric markers and AC species were conducted in obesity or type 2 diabetic populations, and only a few studies have been conducted in normal populations. However, our study was conducted in a general population of Switzerland with a sample size of 2233 participants, the majority of whom were normal-weight participants (62%), and none with obesity.

Our study has several strengths. Firstly, and contrary to most previous studies that were conducted in obesity or type 2 diabetic populations, our study was conducted in the second largest population-based sample of apparently healthy participants in Europe. Secondly, to our knowledge, our study was the second ever prospective study assessing the association between AC and weight changes in adults, and the only one with a follow-up of 10 years. Thirdly, our study excluded participants with obesity or diabetes, which allowed us to examine the associations between the AC species and anthropometric markers without the additional cofounding effect of insulin resistance, antidiabetic treatments, or low-grade chronic inflammation states in the case of obesity. Finally, this is the only study to include both traditional and newer anthropometric indicators for the association with AC species.

However, our study also has several limitations. First, the study was conducted in an apparently healthy population who were relatively lean and metabolically healthy. Therefore, the findings may not be applicable to obese people. Second, as acylcarnitine levels might evolve with time, the results of the prospective study at 10 years should be replicated in other settings. Third, cross-sectional associations in this study may subject to residual confounding factors such as sleep, sleep patterns and menopausal status, despite extensive adjustment for sex, age, smoking, diet, sedentary status within an apparently healthy population-based sample. This limitation may partly explain the different findings observed between cross-sectional and prospective analyses. Fourth, since the present study is an observational study, causal inference cannot be established, and the results should be interpreted with caution. Fifth, some acylcarnitine species, such as adipoylcarnitine, showed higher analytical variability, and the findings of these metabolites should therefore be interpreted with caution. Finally, the participants in our study were recruited from an urban environment with a relatively homogeneous ethnic background, and therefore, the findings may not be able to generalize to other geographic regions or ethnic groups.

## 5. Conclusions

In a sample of apparently healthy, non-obese participants, SCAC and, to a lesser degree, MCAC and LCAC species were positively associated with most anthropometric markers. The associations were similar between sexes, except for MCAC C8:1, which showed a stronger association in females than in males. Specific AC species were prospectively associated with weight gain, suggesting that baseline AC levels may reflect early metabolic alterations linked to adiposity-related metabolic risk.

## Figures and Tables

**Table 1:** Characteristics of the participants at baseline, by sex, CoLaus|PsyCoLaus study, Lausanne, Switzerland.

	Female (n = 1290)	Male (n = 943)	*p*-Value
Age (years)	54.2 ± 8.7	52.3 ± 8.2	<0.001
Smoking categories (%)			0.66
Never	557 (43.2)	390 (41.4)	
Former	438 (34.0)	335 (35.5)	
Current	295 (22.9)	218 (23.1)	
On a diet (%)	323 (25.0)	164 (17.4)	<0.001
Sedentary (%)	724 (56.1)	399 (42.3)	<0.001
Anthropometric markers			
Height (m)	163.9 ± 6.6	176.0 ± 7.2	<0.001
Weight (kg)	62.7 ± 8.9	77.2 ± 9.7	<0.001
Body mass index (kg/m^2^)	23.4 ± 3.0	24.9 ± 2.5	<0.001
BMI categories (%)			
Normal	915 (70.9)	478 (50.7)	<0.001
Overweight	375 (29.1)	465 (49.3)	
Waist (cm)	82.1 ± 9.3	91.0 ± 8.1	<0.001
Abdominal obesity (%)	352 (27.3)	93 (9.9)	<0.001
Hip (cm)	94.5 ± 8.0	97.1 ± 6.4	<0.001
Waist/hip ratio	0.87 ± 0.06	0.94 ± 0.05	<0.001
Waist/height ratio	0.50 ± 0.06	0.52 ± 0.05	<0.001
Bioimpedance (% fat mass)	32.8 ± 6.5	22.3 ± 4.6	<0.001
Conicity index	1.22 ± 0.09	1.26 ± 0.07	<0.001
Body roundness index	3.45 ± 1.13	3.73 ± 0.89	<0.001
Body shape index	7.86 ± 0.53	8.05 ± 0.41	<0.001
Leptin (ng/mL)	4966 [3482–7593]	2784 [1912–4262]	§ <0.001
Adiponectin (μg/mL)	3491 [1566–6478]	744 [358–1569]	§ <0.001
Grip strength (kg)	26.1 ± 6.0	44.8 ± 8.7	<0.001

Results are expressed as average ± standard deviation or median [interquartile range] for continuous variables and as sample size and (column percentage) for categorical variables. Between-group comparisons were performed using *t*-test or Kruskal–Wallis’s test (§) for continuous variables and chi-square for categorical variables.

**Table 2:** Multivariable associations between anthropometric markers and acylcarnitine species, CoLaus|PsyCoLaus study, Lausanne, Switzerland.

		Weight (kg)	BMI (kg/m^2^)	Waist (cm)	Hip (cm)	Waist/Hip Ratio	BIA (% Fat Mass)	Conicity Index	Waist to Heigh Ratio	Body Roundness Index	Body Shape Index	Adiponectin (ng/mL)	Leptin (ng/mL)	Grip Strength (kg)
Short-chain	Deoxy-	0.037	0.002	−0.023	−0.027	−0.010	−0.018	−0.064	−0.052	−0.051	−0.071	−0.019	0.008	0.078
	Acetyl-	−0.052	−0.068	−0.053	−0.062	−0.018	−0.016	−0.025	−0.054	−0.053	−0.004	0.011	**−0.070**	0.015
	Propionyl-	**0.137**	**0.200**	**0.188**	**0.144**	**0.134**	**0.110**	**0.144**	**0.209**	**0.207**	0.091	−0.086	**0.138**	0.014
	Butyryl-	0.041	0.058	0.060	0.043	0.046	0.028	0.052	0.066	0.065	0.037	−0.027	0.077	0.026
	Hydroxybutyryl-	−0.023	−0.024	−0.009	−0.018	0.007	0.015	0.012	−0.005	−0.005	0.022	0.012	−0.039	−0.001
	Glutaryl-	0.068	0.049	0.007	0.012	−0.001	0.023	−0.053	−0.020	−0.020	−0.074	−0.015	0.039	0.032
	Hydroxyvaleryl-	0.011	0.046	0.038	0.027	0.031	0.035	0.038	0.058	0.055	0.026	−0.037	0.038	0.045
	Isovaleryl-	**0.170**	**0.211**	**0.183**	**0.179**	0.090	0.073	0.108	**0.181**	**0.179**	0.048	−0.088	**0.117**	0.046
	Tiglyl-	0.018	0.022	0.001	0.002	0.000	−0.013	−0.018	0.001	−0.002	−0.027	0.008	0.027	0.056
Medium-chain	Adipoyl-	0.056	0.053	0.045	0.054	0.011	0.045	0.016	0.033	0.033	−0.001	0.018	0.038	−0.004
	Hexanoyl-	0.051	0.022	0.033	0.042	0.006	0.017	0.016	0.009	0.007	0.011	0.019	0.007	0.038
	Octanoyl-	0.017	−0.025	−0.010	0.001	−0.017	−0.002	−0.015	−0.039	−0.040	−0.007	0.026	−0.017	0.034
	Octenoyl-	**0.104**	**0.120**	**0.101**	**0.116**	0.033	0.067	0.051	0.093	0.091	0.016	−0.049	0.046	0.015
	Decanoyl-	0.011	−0.038	−0.018	−0.008	−0.018	−0.006	−0.017	−0.049	−0.050	−0.006	0.023	−0.017	0.043
	Decenoyl-	−0.027	−0.079	−0.039	−0.038	−0.019	−0.005	−0.011	−0.064	−0.063	0.014	0.055	−0.026	0.042
	Lauroyl-	−0.006	−0.063	−0.038	−0.032	−0.023	−0.019	−0.029	−0.071	−0.073	−0.010	0.034	−0.029	0.049
	Hydroxydodecanoyl-	−0.045	**−0.104**	−0.073	−0.058	−0.050	−0.026	−0.048	−0.102	−0.100	−0.018	0.025	**−0.056**	0.033
	Dodecenoyl-	0.025	−0.019	0.007	0.012	−0.002	0.014	0.006	−0.023	−0.024	0.013	0.010	−0.019	0.043
Long-chain	Myristolycarnitine	−0.007	−0.075	−0.037	−0.029	−0.026	−0.025	−0.024	−0.078	−0.078	−0.001	0.049	−0.039	0.043
	Hydroxytetradecanoylcarnitine	−0.021	−0.036	−0.017	−0.072	0.050	0.006	0.000	−0.022	−0.022	0.012	−0.003	−0.008	0.035
	Tetradecenoyl-	−0.037	−0.087	−0.062	−0.055	−0.037	−0.015	−0.041	−0.087	−0.087	−0.016	0.050	−0.049	0.038
	Tetradecanedienoyl-	−0.020	−0.063	−0.038	−0.044	−0.012	−0.008	−0.023	−0.061	−0.062	−0.004	0.037	−0.033	0.034
	Palmitoyl-	0.019	−0.001	0.020	0.009	0.021	0.012	0.021	0.005	0.006	0.023	0.020	−0.030	0.045
	Hexadecenoyl-	−0.019	−0.068	−0.046	−0.034	−0.035	−0.008	−0.035	−0.073	−0.073	−0.015	0.079	−0.040	0.022
	Heptadecanoyl-	−0.013	−0.077	−0.052	−0.067	−0.009	−0.052	−0.045	−0.089	−0.089	−0.024	**0.112**	−0.071	0.016
	Stearoyl-	−0.042	**−0.098**	−0.088	−0.089	−0.040	−0.034	−0.079	**−0.118**	**−0.116**	−0.053	0.053	**−0.092**	0.011
	Oleoyl-	−0.039	−0.062	−0.035	−0.037	−0.014	−0.013	−0.009	−0.043	−0.042	0.010	0.007	−0.085	0.014
	Octadecadienoyl-	−0.011	−0.029	−0.004	0.038	−0.045	−0.030	0.013	−0.012	−0.014	0.023	0.028	−0.033	0.026
	Arachidonyl-	−0.004	0.004	0.021	0.021	0.010	−0.002	0.037	0.028	0.028	0.038	0.009	−0.003	0.030
	Free carnitine	**0.111**	**0.175**	**0.152**	**0.126**	0.100	**0.103**	0.110	**0.177**	**0.176**	0.062	−0.064	**0.101**	0.037

BIA, bioimpedance analysis; BMI, body mass index. Results are expressed as multivariable-adjusted beta coefficients. *p*-values are adjusted for multiple comparisons using the False Discovery Rate method of Benjamini–Hochberg and statistically significant coefficients (*p* < 0.001) are indicated in bold. Statistical analysis by linear regression adjusting for sex (male, female), age (continuous), smoking status (never, former, current), presence of a diet (yes, no), and sedentary status (yes, no).

**Table 3:** Multivariable associations between the weight changes over 5 years and 10 years and acylcarnitine species, CoLaus|PsyCoLaus study, Lausanne, Switzerland.

		Weight Changes over 5 Years				Weight Changes over 10 Years			
		Lost 5+ kg	No Change	Gained 5+ kg	*p*-Value	Lost 5+ kg	No Change	Gained 5+ kg	*p*-Value
Short-chain	Deoxy-	759.5 ± 17.6	768.2 ± 4.2	768.5 ± 10.4	0.889	749.2 ± 15.4	770.1 ± 4.8	774.7 ± 9.5	0.364
	Acetyl-	6911 ± 283	7613 ± 68	7516 ± 168	0.052	7552 ± 250	7501 ± 78	7776 ± 154	0.287
	Propionyl-	362.9 ± 12.9	376.9 ± 3.1	383.4 ± 7.6	0.384	386.3 ± 11.5	378.2 ± 3.6	382 ± 7.1	0.738
	Butyryl-	157.7 ± 10.5	181.8 ± 2.5	180.3 ± 6.2	0.083	172.6 ± 9.6	182.3 ± 3	186.8 ± 5.9	0.455
	Hydroxybutyryl-	26.6 ± 2.7	32.3 ± 0.6	33.5 ± 1.6	0.077	32.2 ± 2.4	31.4 ± 0.7	35.6 ± 1.5	0.040
	Glutaryl-	50.1 ± 2.1	50.7 ± 0.5	51.9 ± 1.2	0.622	50.0 ± 1.9	51.5 ± 0.6	51.4 ± 1.2	0.753
	Hydroxyvaleryl-	20.9 ± 1	22.4 ± 0.2	24.3 ± 0.6	**0.001**	22.0 ± 0.8	22.3 ± 0.3	24.1 ± 0.5	**0.007**
	Isovaleryl-	85.8 ± 3.8	90.4 ± 0.9	93.2 ± 2.3	0.228	90.7 ± 3.4	91.1 ± 1.1	95.1 ± 2.1	0.229
	Tiglyl-	11.4 ± 0.6	12.2 ± 0.2	12.5 ± 0.4	0.326	12.0 ± 0.6	12.4 ± 0.2	12.5 ± 0.4	0.754
Medium-chain	Adipoyl-	23.3 ± 1.5	22 ± 0.3	23.1 ± 0.9	0.405	23.9 ± 1.3	22.1 ± 0.4	22.7 ± 0.8	0.338
	Hexanoyl-	35.8 ± 3.9	38.7 ± 0.9	37.4 ± 2.3	0.680	38.2 ± 3.6	37.3 ± 1.1	42.8 ± 2.2	0.098
	Octanoyl-	114.6 ± 17.6	129 ± 4.2	117.5 ± 10.4	0.464	120 ± 16.4	123.6 ± 5.1	140.5 ± 10.2	0.311
	Octenoyl-	67.8 ± 5	72.3 ± 1.2	79.1 ± 3.0	0.063	68.2 ± 4.4	71.3 ± 1.4	81 ± 2.7	**0.004**
	Decanoyl-	190.9 ± 25	214.2 ± 6	195.2 ± 14.8	0.361	200.1 ± 23.3	206.9 ± 7.3	228.3 ± 14.4	0.381
	Decenoyl-	76.1 ± 4	78.1 ± 1	75.0 ± 2.4	0.443	79.4 ± 3.6	76.7 ± 1.1	79.7 ± 2.2	0.416
	Lauroyl-	56.5 ± 4.9	62.0 ± 1.2	58.4 ± 2.9	0.325	60.4 ± 4.5	60.4 ± 1.4	65.1 ± 2.8	0.333
	Hydroxydodecanoyl-	11.4 ± 0.7	12.3 ± 0.2	11.6 ± 0.4	0.137	12.7 ± 0.6	12.1 ± 0.2	12.2 ± 0.4	0.708
	Dodecenoyl-	100.3 ± 5.5	107.5 ± 1.3	102.5 ± 3.3	0.190	104.7 ± 5	105.9 ± 1.6	108.8 ± 3.1	0.675
Long-chain	Myristolycarnitine	25.3 ± 1.3	26.3 ± 0.3	25.7 ± 0.7	0.574	27.1 ± 1.1	25.8 ± 0.4	27.1 ± 0.7	0.166
	Hydroxytetradecanoylcarnitine	5.8 ± 0.4	5.9 ± 0.1	5.8 ± 0.2	0.957	6.3 ± 0.4	5.7 ± 0.1	5.9 ± 0.2	0.171
	Tetradecenoyl-	67.5 ± 4.5	73.5 ± 1.1	70 ± 2.6	0.234	72.6 ± 4	71.7 ± 1.3	76.3 ± 2.5	0.263
	Tetradecanedienoyl-	44.4 ± 2.8	49.8 ± 0.7	48.5 ± 1.7	0.166	48.4 ± 2.5	49 ± 0.8	51.1 ± 1.6	0.460
	Palmitoyl-	117.4 ± 3	118.2 ± 0.7	118.5 ± 1.8	0.948	122.7 ± 2.7	117 ± 0.8	120.5 ± 1.7	**0.033**
	Hexadecenoyl-	29.6 ± 1.4	30.3 ± 0.3	29.9 ± 0.8	0.804	31.5 ± 1.2	29.6 ± 0.4	31.3 ± 0.8	0.082
	Heptadecanoyl-	4.5 ± 0.2	4.4 ± 0.1	4.2 ± 0.1	0.336	4.6 ± 0.2	4.4 ± 0.1	4.3 ± 0.1	0.406
	Stearoyl-	48.2 ± 1.7	49.2 ± 0.4	49 ± 1	0.860	50.8 ± 1.5	48.8 ± 0.5	49.8 ± 0.9	0.376
	Oleoyl-	181.4 ± 6.9	183 ± 1.7	182 ± 4.1	0.956	188.5 ± 6.1	180.3 ± 1.9	187.2 ± 3.8	0.144
	Octadecadienoyl-	53.9 ± 3.8	55.9 ± 0.9	54.5 ± 2.2	0.777	63.9 ± 3.4	54.2 ± 1.1	58.6 ± 2.1	**0.007**
	Arachidonyl-	5.3 ± 0.3	5.4 ± 0.1	5.5 ± 0.2	0.688	5.4 ± 0.3	5.3 ± 0.1	5.6 ± 0.2	0.250
	Free carnitine	34.4 ± 0.8	34.6 ± 0.2	35.1 ± 0.5	0.487	34.7 ± 0.7	34.5 ± 0.2	35 ± 0.4	0.658

Values are expressed as model-adjusted means of baseline acylcarnitine concentrations ± standard errors across three weight change categories (≥5 kg weight loss, stable weight, ≥5 kg weight gain) over 5-year and 10-year follow-up. Significant *p*-values are indicated in bold. Statistical analysis was performed by multivariable ANOVA models adjusting for sex (male, female), age (continuous), smoking status (never, former, current), presence of a diet (yes, no), and sedentary status (yes, no).

## Data Availability

The data of the CoLaus|PsyCoLaus study used in this article cannot be fully shared as they contain potentially sensitive personal information on participants. According to the Ethics Committee for Research of the Canton of Vaud, sharing these data would be a violation of the Swiss legislation with respect to privacy protection. However, coded individual-level data that do not allow researchers to identify participants are available upon request to researchers who meet the criteria for data sharing of the CoLaus|PsyCoLaus Datacentre (CHUV, Lausanne, Switzerland). Any researcher affiliated with a public or private research institution who complies with the CoLaus|PsyCoLaus standards can submit a research application to research.colaus@chuv.ch or research.psycolaus@chuv.ch. Proposals requiring baseline data only, will be evaluated by the baseline (local) Scientific Committee (SC) of the CoLaus and PsyCoLaus studies. Proposals requiring follow-up data will be evaluated by the follow-up (multicentric) SC of the CoLaus|PsyCoLaus cohort study. Detailed instructions for gaining access to the CoLaus|PsyCoLaus data used in this study are available at www.colaus-psycolaus.ch/professionals/how-to-collaborate/ (accessed on 1 May 2025).

## Supplementary Materials

The following supporting information can be downloaded at https://www.mdpi.com/article/10.3390/metabo16050315/s1. Table S1: Acylcarnitine species nomenclature. Table S2: Internal standards of the metabolites included in the analysis. Table S3: Characteristics of the included and excluded participants, CoLaus|PsyCoLaus study, Lausanne, Switzerland. Table S4: Bivariate associations between anthropometric markers and acylcarnitine species, CoLaus|PsyCoLaus study, Lausanne, Switzerland. Table S5: Bivariate associations between the weight gain ≥ 5 kg over 5 years and 10 years and acylcarnitine species, CoLaus|PsyCoLaus study, Lausanne, Switzerland. Table S6: Levels of acylcarnitine species between male and female participants, CoLaus|PsyCoLaus study, Lausanne, Switzerland. Table S7: Multivariable associations between anthropometric markers and acylcarnitine species, stratified by sex, CoLaus|PsyCoLaus study, Lausanne, Switzerland. Table S8: Levels of acylcarnitine species between pre- and post-menopausal female participants, CoLaus|PsyCoLaus study, Lausanne, Switzerland. Table S9: Multivariable associations between anthropometric markers and acylcarnitine species, after excluding early weight gainers (≥5 kg at first follow-up), CoLaus|PsyCoLaus study, Lausanne, Switzerland. Table S10: Multivariable-adjusted differences in acylcarnitine concentrations associated with ≥5 kg weight loss or ≥5 kg weight gain compared with stable weight, CoLaus|PsyCoLaus study, Lausanne, Switzerland. Figure S1: Selection procedure. Figure S2: Spike graph showing the beta correlation coefficients between acylcarnitine species and anthropometry markers, stratified by sex. The X-axis indicate the absolute value of the beta correlation coefficients. Red colour indicates a negative association, black colour indicates a positive association. Figure S3: Beta coefficients representing the associations between anthropometric markers and acylcarnitine species.

## Abbreviations

The following abbreviations are used in this manuscript:

ABSI	A Body Shape Index
AC	Acylcarnitine
ACN	Acetonitrile
BIA	Bioelectrical impedance analyses
BMI	Body Mass Index
BRI	Body roundness index
CI	Conicity Index
FU	Follow-up
HESI	Heated electrospray ionization
LC-HRMS	Liquid Chromatography–High Resolution Mass Spectrometry Analysis
LC-MS	Liquid Chromatography–Mass Spectrometry
LCAC	Long-chained acylcarnitine
MCAC	Medium-chain acylcarnitine
SCAC	Short-chain acylcarnitine
T2DM	Type 2 Diabetes Mellitus
UHPLC	Ultrahigh-performance liquid chromatography
WC	Waist circumference
WHO	World Health Organization
