# Supplementary material for: Association of Acylcarnitine Species and Anthropometry Markers in a Population-Based Apparently Healthy Cohort
Source: Metabolites. 2026 May 6;16(5):315. doi: 10.3390/metabo16050315 (PMC13208298; doi:10.3390/metabo16050315)
Supplement: Supplementary file 1 [file metabolites-16-00315-s001.zip › metabolites-4271535-supplementary tables.pdf]

Supplementary table S1: Acylcarnitine species nomenclature

	Acylcarnitine species (Full name)	Chain-length class (functional group)
-	Deoxycarnitine	Precursor of carnitine
C2:0	Acetylcarnitine	Short-chain
C3:0	Propionylcarnitine	Short-chain
C4:0	Butyrylcarnitine	Short-chain
C4-OH	Hydroxybutyrylcarnitine	Short-chain
C5:0-DC	Glutarylcarnitnie	Short-chain
C5:0-OH	Hydroxyvalerylcarnitine	Short-chain
C5:0 I	Isovalerylcarnitine	Short-chain
C5:1 M	Tiglylcarnitine	Short-chain
C6:0-DC	Adipoylcarnitine	Medium-chain
C6-OH	Hexanoylcarnitine	Medium-chain
C8:0	Octanoylcarnitine	Medium-chain
C8:1	Octenoylcarnitine	Medium-chain
C10:0	Decanoylcarnitine	Medium-chain
C10:1	Decenoylcarnitine	Medium-chain
C12:0	Lauroylcarnitine	Medium-chain
C12:0	Hydroxydodecanoyl-carnitine	Medium-chain
C12:1	Dodecenoyl-carnitne	Medium-chain
C14:0	Myristolycarnitne	Long-chain
C14:0	Hydroxytetradecanoyl-carnitine	Long-chain
C14:1	Tetradecenoyl-carnitine	Long-chain
C14:2	Tetradecanedienoyl-carnitine	Long-chain
C16:0	Palmitoylcarnitine	Long-chain
C16:1	Hexadecenoyl-carnitine	Long-chain
C17:0	Heptadecanoylcarnitine	Long-chain
C18:0	Stearoylcarnitine	Long-chain
C18:1	Oleoyl-carnitine	Long-chain
C18:2	Octadecadienoyl-carnitine	Long-chain
C20:4	Arachidonyl-carnitine	Long-chain
Total	Carnitine	Free carnitine

Based on the References.

- Human Metabolome Database
- Dambrova M, Makrecka-Kuka M, Kuka J, Vilskersts R, Nordberg D, Attwood MM, et al. Acylcarnitines: Nomenclature, Biomarkers, Therapeutic Potential, Drug Targets, and Clinical Trials. Pharmacol Rev. 2022 Jul;74(3):506–51, https://doi.org/10.1124/pharmrev.121.000408

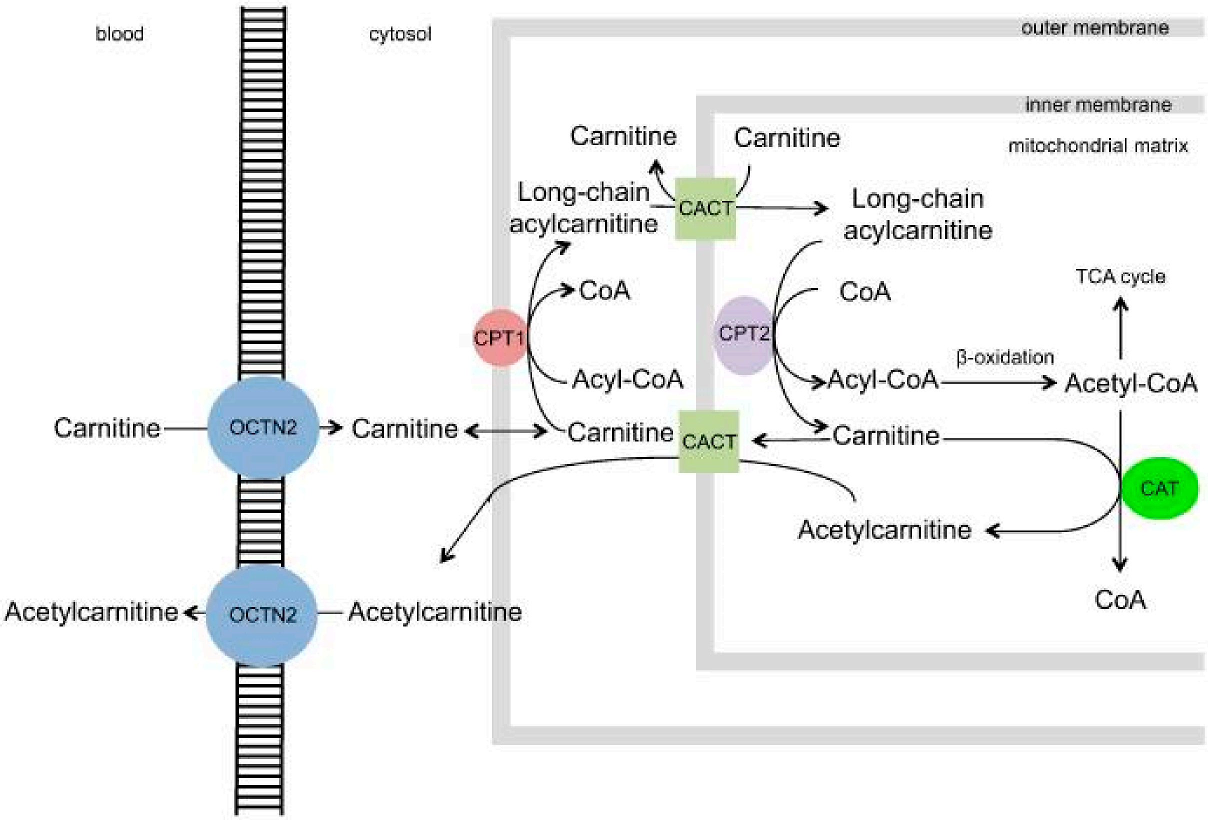

The carnitine shuttle.

Carnitine enters the cell through active transport by the high affinity carnitine transporter, organic cation transporter novel 2 (OCTN2). Long-chain fatty acid-CoA in the cytosol exchanges CoA for carnitine by the action of carnitine palmitoyltransferase I (CPT1) in the outer mitochondrial membrane. Acylcarnitine moves into the mitochondrial matrix by facilitated diffusion through a transporter, carnitine-acylcarnitine translocase (CACT), on the inner mitochondrial membrane, in exchange for carnitine. In the mitochondrial matrix, the acyl group is transferred to mitochondrial coenzyme A by carnitine palmitoyltransferase II (CPT2). Carnitine is then free to cycle back to the cytosol through the transporter. Carnitine acyltransferase (CAT) removes CoA from acetyl-CoA that is formed from β-oxidation to form acetylcarnitine. Acetylcarnitine can exit the mitochondria via CACT and enter the blood via OCTN2.

Carnitine biosynthesis:

Lysine → Trimethyllysine → Deoxycarnitine → Carnitine

Source:

https://pmc.ncbi.nlm.nih.gov/articles/PMC5360565/

https://medicoapps.org/m-carnitine/

Supplementary table S2. Internal standards of the metabolites included in the analysis.

**Metabolite**	**Abbreviation**	**ISTD type**	**Associated ISTD**	**CV (internal pool QC)**
deoxycarnitine	Deoxy-C0	analogue	γ-Butyrobetaine-d9	4.9
carnitine	C0	**authentic**	L-Carnitine-(N-methyl-d3), inner salt	2.5
acetylcarnitine	C2	**authentic**	Acetyl-L-carnitine-(N-methyl-d3)	2.6
propionylcarnitine	C3	**authentic**	Propionyl-L-carnitine-(N-methyl-d3)	3.1
butyrylcarnitine	C4/iC4	**authentic**	Butyryl-L-carnitine-(N-methyl-d3)	3.6
hydroxybutyrylcarnitine	*C4-OH*	***authentic***	*Hydroxybutyrylcarnitine-d3*	***21.9***
isovalerylcarnitine	C5/iC5	**authentic**	Isovaleryl-L-carnitine-(N,N,N-trimethyl-d9)	6.8
tiglylcarnitine	*C5:1*	***authentic***	*Tiglylcarnitine-d3*	***37.8***
glutarylcarnitine	C5-DC	**authentic**	Glutaryl-L-carnitine-(N-methyl-d3) lithium salt	18.8
hydroxyvalerylcarnitine	*iC5-OH*	***authentic***	*3-Hydroxyisovaleryl-L-carnitine-(N-methyl-d3)*	*20.8*
hexanoylcarnitine	C6	**authentic**	Hexanoyl-L-carnitine-(N-methyl-d3)	4.5
adipoylcarnitine	*C6-DC*	***authentic***	*adipoylcarnitine-d3*	*20.9*
octanoylcarnitine	C8	**authentic**	Octanoyl-L-carnitine-(N-methyl-d3)	4.4
octenoylcarnitine	C8:1	analogue	Octanoyl-L-carnitine-(N-methyl-d3)	3.9
decanoylcarnitine	C10	**authentic**	Decanoyl-L-carnitine-(N-methyl-d3)	4.8
decenoylcarnitine	C10:1	analogue	Decanoyl-L-carnitine-(N-methyl-d3)	3.6
lauroylcarnitine	C12	**authentic**	Lauroyl-L-carnitine-(N,N,N-trimethyl-d9)	5.5
dodecenoylcarnitine	C12:1	analogue	Lauroyl-L-carnitine-(N,N,N-trimethyl-d9)	4.1
hydroxydodecanoylcarnitine	*C12-OH*	*analogue*	*Lauroyl-L-carnitine-(N,N,N-trimethyl-d9)*	***74.5***
myristoylcarnitine	C14	**authentic**	Myristoyl-L-carnitine-(N,N,N-trimethyl-d9)	13.1
tetradecenoylcarnitine	C14:1	analogue	Myristoyl-L-carnitine-(N,N,N-trimethyl-d9)	8.3
tetradecanedienoylcarnitine	C14:2	analogue	Myristoyl-L-carnitine-(N,N,N-trimethyl-d9)	6.4
hydroxytetradecanoylcarnitine	*C14-OH*	*analogue*	*[(R)-3-Hydroxyhexadecanoyl]-L-carnitine-(methyl-d3)*	***47.3***
palmitoylcarnitine	C16	**authentic**	Palmitoyl-L-carnitine-(N-methyl-d3)	6.6
hexadecenoylcarnitine	C16:1	analogue	Palmitoyl-L-carnitine-(N-methyl-d3)	13.8

heptadecanoylcarnitine	*C17*	*analogue*	*Palmitoyl-L-carnitine-(N-methyl-d3)*	***96.3***
stearoylcarnitine	C18	**authentic**	Stearoyl-L-carnitine-(N-methyl-d3)	16.9
oleoylcarnitine	C18:1	**authentic**	Oleoyl-L-carnitine-d3 Inner Salt	5.8
octadecadienoylcarnitine	C18:2	analogue	Stearoyl-L-carnitine-(N-methyl-d3)	6.8
arachidonylcarnitine	*C20:4*	*analogue*	*Stearoyl-L-carnitine-(N-methyl-d3)*	***68.6***

Supplementary table S3: Characteristics of the included and excluded participants, CoLaus|PsyCoLaus study, Lausanne, Switzerland.

	**Included**<br>**N=2,233**	**Excluded**<br>**N=349**	**p-value**
Male sex (%)	943 (42.2)	158 (45.3)	0.29
Age (years)	53.4±8.6	53.7±8.6	0.57
Smoking status (%)			<0.001
Never	947 (42.4)	133 (41.2)	
Former	773 (34.6)	83 (25.7)	
Current	513 (23.0)	107 (33.1)	
Alcohol consumption (%)	1,767 (79.1)	233 (66.8)	<0.001
Any diet (%)	487 (21.8)	65 (18.6)	0.18
Sedentary status (%)	1,123 (50.3)	3 (42.9)	0.69
Grip strength (kg)	34.1±11.8	33.8±11.5	0.70
Body mass index (kg/m^2)	24.0±2.9	24.6±3.2	<0.001
Waist (cm)	85.8±9.8	88.0±10.1	<0.001
Abdominal obesity (%)	445 (19.9)	84 (24.3)	0.063
Adiponectin (ng/mL)	3955 [2535-6260]	4336 [2643-6098]	§ 0.27
Leptin (ng/mL)	1833 [678-4434]	2025 [985-4474]	§ 0.067

Results are expressed as number of participants (column percentage) for categorical variables and as mean standard deviation for continuous variables. Between group comparisons performed using chi-square for categorical variables and student's t-test or Kruskal-Wallis test (§) for continuous variables. Due to missing values, the numbers in the excluded column might not add to total.

Supplementary table S4. Bivariate associations between anthropometric markers and acylcarnitine species, CoLaus|PsyCoLaus study, Lausanne, Switzerland.

		Weight (kg)	BMI (kg/m^2)	Waist (cm)	Hip (cm)	Waist/hip ratio	BIA (% fat mass)	Conicity index	Waist to heigh ratio	Body roundness index	Body shape index	Adiponectin (ng/mL)	Leptin (ng/mL)	Grip strength (kg)
	Deoxy-	**0.343**	**0.140**	**0.217**	**0.093**	**0.241**	**-0.364**	**0.090**	0.046	0.046	0.049	**-0.222**	**-0.262**	**0.452**
	Acetyl-	-0.063	-0.065	-0.038	-0.037	-0.029	0.050	0.003	-0.036	-0.036	0.027	0.077	-0.083	-0.040
	Propionyl-	**0.346**	**0.280**	**0.343**	**0.219**	**0.298**	**-0.145**	**0.246**	**0.263**	**0.263**	**0.175**	**-0.229**	-0.045	**0.282**
	Butyryl-	**0.139**	**0.122**	**0.142**	**0.103**	**0.108**	-0.015	**0.096**	**0.118**	**0.118**	0.062	-0.085	0.047	**0.106**
Short-chain	Hydroxybutyryl-	0.028	0.002	0.045	0.024	0.045	0.027	0.059	0.023	0.023	0.068	0.025	**-0.096**	0.029
	Glutaryl-	**0.215**	**0.119**	**0.126**	0.059	**0.134**	**-0.140**	0.027	0.036	0.036	-0.010	**-0.146**	-0.080	**0.221**
	Hydroxyvaleryl-	**0.152**	**0.133**	**0.150**	**0.096**	**0.132**	**-0.099**	**0.099**	**0.119**	**0.119**	0.065	**-0.110**	-0.064	**0.191**
	Isovaleryl-	**0.437**	**0.318**	**0.379**	**0.259**	**0.317**	**-0.260**	**0.231**	**0.251**	**0.252**	**0.144**	**-0.277**	**-0.134**	**0.387**
	Tiglyl-	**0.148**	0.086	**0.118**	0.062	**0.115**	**-0.134**	0.068	0.063	0.063	0.046	-0.079	**-0.110**	**0.193**
	Adipoyl-	0.003	0.046	0.011	0.069	-0.056	**0.173**	-0.013	0.034	0.034	-0.029	**0.114**	**0.148**	**-0.126**
	Hexanoyl-	0.079	0.060	**0.125**	**0.099**	0.086	0.065	**0.133**	**0.106**	**0.106**	**0.125**	-0.002	0.006	0.025
	Octanoyl-	-0.019	-0.061	0.005	0.004	0.004	0.049	0.045	-0.017	-0.017	0.068	0.024	-0.041	0.003
	Octenoyl-	**0.156**	**0.150**	**0.154**	**0.160**	0.073	0.043	**0.095**	**0.129**	**0.129**	0.053	-0.079	0.020	0.058
Medium-chain	Decanoyl-	-0.046	**-0.106**	-0.031	-0.034	-0.012	0.022	0.019	-0.062	-0.062	0.055	0.033	-0.076	0.014
	Decenoyl-	-0.037	-0.080	-0.008	-0.016	0.002	0.042	0.042	-0.033	-0.033	0.072	0.070	-0.078	-0.004
	Lauroyl-	0.034	-0.085	0.017	-0.019	0.049	**-0.094**	0.043	-0.064	-0.064	0.077	-0.011	**-0.175**	**0.124**
	Hydroxydodecanoyl-	0.038	-0.061	0.018	0.012	0.017	-0.067	0.032	-0.051	-0.051	0.055	-0.007	**-0.178**	**0.091**
	Dodecenoyl-	0.042	-0.003	0.061	0.045	0.045	0.030	0.073	0.030	0.030	0.075	-0.017	-0.044	0.049
	Myristolycarnitine	0.046	-0.061	0.037	0.012	0.047	-0.063	0.062	-0.038	-0.038	**0.092**	0.028	**-0.137**	0.083
	Hydroxytetradecanoylcarnitine	**0.102**	0.007	**0.095**	0.010	**0.141**	-0.085	**0.099**	0.025	0.025	**0.107**	-0.069	**-0.147**	**0.139**
	Tetradecenoyl-	-0.024	**-0.096**	-0.025	-0.043	0.003	-0.022	0.013	-0.070	-0.070	0.047	0.036	**-0.142**	0.043
	Tetradecanedienoyl-	0.025	-0.053	0.022	-0.018	0.048	-0.046	0.040	-0.031	-0.031	0.058	-0.011	**-0.153**	0.082
Long-chain	Palmitoyl-	**0.158**	0.074	**0.157**	**0.087**	**0.152**	**-0.111**	**0.137**	0.085	0.085	**0.126**	-0.048	**-0.151**	**0.172**
	Hexadecenoyl-	**-0.106**	**-0.102**	-0.062	-0.035	-0.068	**0.137**	0.005	-0.050	-0.050	0.042	**0.161**	-0.030	**-0.132**
	Heptadecanoyl-	0.022	-0.055	0.001	-0.026	0.031	-0.043	0.018	-0.047	-0.047	0.041	**0.108**	**-0.097**	0.026
	Stearoyl-	**0.104**	-0.025	0.058	-0.010	**0.098**	**-0.163**	0.049	-0.034	-0.034	0.062	-0.008	**-0.221**	**0.152**
	Oleoyl-	-0.010	-0.050	0.019	-0.003	0.026	-0.003	0.063	-0.004	-0.004	0.084	0.042	**-0.130**	0.004

Octadecadienoyl-	0.062	-0.009	0.055	0.069	0.016	**-0.101**	0.058	0.006	0.006	0.063	-0.018	**-0.122**	**0.114**
Arachidonyl-	**0.256**	**0.117**	**0.225**	**0.109**	**0.239**	**-0.274**	**0.174**	**0.112**	**0.112**	**0.146**	**-0.146**	**-0.236**	**0.315**
Free-carnitine	**0.245**	**0.219**	**0.259**	**0.163**	**0.225**	-0.082	**0.193**	**0.214**	**0.214**	**0.136**	**-0.164**	-0.031	**0.206**

BIA, bioimpedance analysis; BMI, body mass index. Results are expressed as Spearman rank correlation coefficients. P-values are adjusted for multiple comparisons using the False Discovery Rate method of Benjamini-Hochberg and statistically significant coefficients (p<0.001) are indicated in bold.

Supplementary table S5. Bivariate associations between the weight gain ≥ 5 kg over 5 years and 10 years and acylcarnitine species, CoLaus|PsyCoLaus study, Lausanne, Switzerland.

		Weight changes over 5 years				Weight changes over 10 years			
		Lost 5+kg	No change	Gained 5+ kg	p-value	Lost 5+kg	No change	Gained 5+ kg	p-value
	Deoxy-	794.3 (656.6-864.5)	760.4 (638.8-894.0)	740.6 (629.5-860.6)	0.38	744.1 (644.1-865.9)	756.7 (638.8-887.4)	744.2 (637.6-853.3)	0.50
	Acetyl-	7195.1 (5990.9-9309.0)	7020.5 (5655.3-8795.4)	6855.1 (5504.8-9000.2)	0.38	6376.8 (4899.2-8304.8)	7157.0 (5716.1-8978.1)	6686.0 (5367.6-8574.5)	**<0.05**
	Propionyl-	393.0 (311.4-465.0)	359.6 (285.2-444.9)	359.4 (282.0-433.9)	0.14	349.1 (273.5-428.0)	360.7 (287.5-445.1)	356.5 (283.3-427.5)	0.58
	Butyryl-	155.9 (114.4-210.9)	164.2 (127.8-213.0)	159.5 (127.1-210.4)	0.38	147.2 (109.6-191.2)	162.9 (126.0-212.2)	155.8 (128.5-205.6)	**<0.05**
Short-chain	Hydroxybutyryl-	27.6 (18.9-46.3)	25.4 (17.8-37.3)	25.3 (17.3-38.9)	0.21	21.5 (15.0-34.5)	25.9 (17.9-38.8)	24.5 (16.6-35.2)	**<0.05**
	Glutaryl-	48.5 (39.1-59.0)	48.8 (37.8-61.9)	47.1 (36.5-61.6)	0.65	45.8 (35.2-59.8)	47.9 (37.5-60.9)	48.0 (37.1-62.4)	0.94
	Hydroxyvaleryl-	21.9 (17.5-26.4)	21.4 (17.1-26.4)	21.1 (17.0-27.0)	0.78	20.4 (16.8-25.6)	21.4 (17.1-26.3)	21.7 (17.2-26.4)	0.27
	Isovaleryl-	87.1 (67.4-115.1)	84.0 (66.3-108.8)	83.4 (64.3-108.4)	0.45	81.8 (63.4-107.0)	83.5 (65.9-108.3)	83.6 (65.9-107.0)	0.63
	Tiglyl-	12.1 (7.9-16.8)	11.6 (8.1-15.6)	10.8 (7.5-14.5)	0.18	10.3 (6.6-15.3)	11.4 (8.0-15.4)	10.9 (7.5-15.1)	0.26
Me	Adipoyl-	21.5 (15.9-27.6)	19.0 (14.4-26.7)	19.4 (14.4-26.2)	0.095	20.9 (15.4-28.3)	19.2 (14.6-26.7)	18.9 (13.9-25.2)	0.33

	Hexanoyl-	35.5 (27.9-47.9)	33.9 (26.8-43.5)	33.5 (25.4-44.4)	0.13	32.5 (25.7-40.8)	34.5 (27.2-44.5)	32.1 (24.5-42.0)	**<0.05**
	Octanoyl-	106.6 (78.7-148.6)	106.5 (77.9-144.6)	98.0 (73.3-140.8)	0.073	99.9 (71.3-138.4)	106.9 (78.8-147.8)	96.3 (72.7-134.5)	**<0.05**
	Octenoyl-	61.0 (40.0-90.7)	60.6 (41.0-87.2)	63.6 (43.7-94.8)	0.18	48.9 (34.5-84.5)	61.6 (42.5-88.4)	61.3 (41.7-92.9)	0.066
	Decanoyl-	179.1 (131.8-253.3)	179.2 (131.4-243.1)	162.8 (119.8-241.6)	**<0.05**	162.0 (117.1-241.4)	180.1 (131.9-248.0)	158.2 (118.6-232.4)	**<0.001**
	Decenoyl-	77.3 (53.8-106.5)	70.3 (54.0-95.2)	65.3 (49.0-88.3)	**<0.05**	67.4 (44.5-97.3)	71.8 (54.1-96.0)	63.3 (47.8-85.2)	**<0.001**
	Lauroyl-	58.8 (41.8-79.4)	54.2 (39.8-74.0)	49.6 (36.4-68.8)	**<0.05**	48.4 (36.3-75.1)	54.8 (39.8-75.1)	49.3 (35.4-65.5)	**<0.001**
	Hydroxydodecanoyl-	12.3 (8.0-17.9)	11.2 (7.5-15.4)	9.8 (6.4-14.5)	**<0.05**	9.3 (6.2-14.5)	11.3 (7.6-15.7)	9.0 (6.2-13.9)	**<0.001**
	Dodecenoyl-	102.4 (77.8-130.3)	99.2 (75.5-126.5)	95.2 (70.9-122.6)	0.12	96.1 (69.9-116.4)	99.9 (76.4-128.6)	90.2 (70.5-117.8)	**<0.05**
	Myristolycarnitine	27.8 (21.0-33.6)	24.3 (19.4-31.0)	23.2 (18.1-29.5)	**<0.001**	23.8 (17.8-31.3)	24.7 (19.4-31.4)	22.6 (18.1-28.5)	**<0.05**
	Hydroxytetradecanoylcarnitine	5.8 (3.2-8.7)	4.9 (3.0-7.2)	4.4 (2.9-7.0)	**<0.05**	4.6 (2.3-7.8)	5.0 (3.1-7.5)	4.4 (2.6-6.5)	**<0.05**
	Tetradecenoyl-	70.8 (48.8-95.2)	64.6 (47.1-88.4)	59.4 (43.8-85.2)	**<0.05**	55.5 (42.5-87.7)	65.6 (47.2-90.5)	60.3 (41.4-81.7)	**<0.05**
Long-chain	Tetradecanedienoyl-	45.1 (32.5-61.5)	44.5 (32.4-61.3)	41.7 (29.7-57.1)	0.075	37.4 (26.6-57.6)	44.8 (32.9-61.5)	40.5 (29.2-53.5)	**<0.001**
	Palmitoyl-	121.7 (105.1-149.8)	115.0 (95.7-136.2)	114.4 (92.1-134.4)	**<0.05**	111.5 (97.2-140.8)	116.3 (97.2-136.7)	112.4 (93.3-132.2)	0.14
	Hexadecenoyl-	29.9 (21.8-40.5)	28.2 (21.5-35.7)	26.0 (20.4-34.6)	**<0.05**	27.5 (19.7-36.7)	28.5 (21.8-36.7)	26.5 (20.2-34.3)	**<0.05**
	Heptadecanoyl-	4.6 (3.5-6.0)	4.2 (2.9-5.6)	3.8 (2.7-5.3)	**<0.05**	4.4 (2.9-5.9)	4.2 (2.9-5.7)	3.8 (2.6-5.3)	**<0.05**
	Stearoyl-	49.9 (39.8-62.9)	47.1 (37.0-58.8)	44.8 (35.6-58.1)	**<0.05**	46.9 (34.7-60.9)	47.3 (37.5-58.9)	43.3 (34.9-57.7)	**<0.05**
	Oleoyl-	181.9 (145.9-229.5)	172.8 (137.7-211.6)	165.2 (132.6-215.8)	0.081	167.6 (128.3-224.8)	173.2 (139.1-214.7)	165.5 (130.1-208.8)	0.12

Octadecadienoyl-	49.0 (42.2-69.6)	47.4 (37.7-61.2)	46.7 (37.0-64.2)	0.11	45.6 (35.6-61.1)	47.8 (38.3-62.1)	45.2 (36.4-60.3)	0.089
Arachidonyl-	5.1 (3.3-7.4)	5.0 (3.0-7.2)	5.0 (3.0-7.0)	0.43	4.4 (3.0-6.9)	5.0 (3.1-7.2)	4.9 (3.1-7.0)	0.65
Free-carnitine	34.9 (30.4-39.2)	34.0 (29.4-39.6)	34.0 (29.1-39.0)	0.47	34.3 (28.8-38.8)	34.2 (29.7-39.5)	34.1 (28.8-39.5)	0.83

Values are expressed as means of baseline acylcarnitine concentrations with interquartile ranges across ≥5 kg weight gain over 5-year and 10-year follow-ups. Significant p-values are indicated in bold. Statistical analysis by Wilcoxon rank-sum test.

Supplementary table S6. Levels of acylcarnitine species between male and female participants, CoLaus|PsyCoLaus study, Lausanne, Switzerland.

		Female N=1,290	Male N=943	p-value
Short chain	Deoxycarnitine	675.9 [584.1-778.9]	863.2 [755.9-981.2]	<0.001
	Acetylcarnitine	7050 [5655-8998]	6962 [5618-8774]	0.61
	Propionylcarnitine	323.2 [262.3-396.0]	414.0 [340.4-501.7]	<0.001
	Butyrylcarnitine	155.8 [119.7-202.7]	170.1 [130.6-227.2]	<0.001
	Hydroxybutyrylcarnitine	24.2 [16.8-37.1]	26.9 [18.7-40.5]	<0.001
	Glutarylcarnitine	44.5 [34.5-55.9]	52.9 [41.7-66.9]	<0.001
	Hydroxyvalerylcarnitine	20.3 [15.9-24.7]	23.3 [18.9-28.2]	<0.001
	Isovalerylcarnitine	72.4 [58.1-89.8]	104.1 [83.4-127.8]	<0.001
	Tiglylcarnitine	10.3 [7.1-14.2]	12.7 [9.2-16.8]	<0.001
Medium-chain	Adipoylcarnitine	20.1 [14.9-28.1]	18.2 [14.0-23.8]	<0.001
	Hexanoylcarnitine	33.5 [26.2-43.8]	35.5 [28.1-45.3]	<0.001
	Octanoylcarnitine	102.6 [77.4-143.9]	106.6 [78.5-147.4]	0.14
	Octenoylcarnitine	58.9 [39.2-85.9]	62.5 [44.1-93.7]	<0.001
	Decanoylcarnitine	174.6 [126.3-243.6]	180.1 [130.6-249.3]	0.092
	Decenoylcarnitine	69.7 [51.5-93.4]	71.2 [53.3-97.2]	0.092
	Lauroylcarnitine	50.2 [37.0-68.9]	58.1 [42.3-81.7]	<0.001
	Hydroxydodecanoylcarnitine	10.3 [6.8-14.4]	12.1 [8.0-17.4]	<0.001
	Dodecenoylcarnitine	96.2 [73.2-123.5]	101.8 [78.0-131.0]	<0.001
Long-chain	Myristolycarnitine	23.3 [18.3-29.5]	26.1 [20.4-33.2]	<0.001
	Hydroxytetradecanoylcarnitine	4.4 [2.6-6.6]	5.7 [3.7-8.5]	<0.001
	Tetradecenoylcarnitine	62.6 [45.2-85.6]	67.3 [48.7-94.9]	<0.001
	Tetradecanedienoylcarnitine	41.4 [30.4-56.7]	47.3 [34.5-66.1]	<0.001
	Palmitoylcarnitine	109.7 [91.3-128.8]	124.0 [104.9-147.6]	<0.001
	Hexadecenoylcarnitine	29.0 [22.3-37.1]	27.1 [20.8-34.6]	<0.001
	Heptadecanoylcarnitine	4.0 [2.8-5.5]	4.3 [3.0-5.9]	0.002
	Stearoylcarnitine	43.4 [34.3-55.3]	51.4 [41.3-63.9]	<0.001
	Oleoylcarnitine	169.9 [134.5-210.9]	175.9 [140.9-220.9]	0.001
	Octadecadienoylcarnitine	44.8 [35.5-57.9]	50.9 [40.8-65.6]	<0.001
	Arachidonylcarnitine	3.9 [2.5-5.8]	6.5 [4.6-8.7]	<0.001
	Free carnitine	32.5 [27.8-37.8]	36.8 [31.8-41.9]	<0.001

Results are expressed as median [interquartile range]. Statistical analysis by Wilcoxon rank-sum test. All values in nMol except free carnitine (μMol).

Supplementary table S7. Multivariable associations between anthropometric markers and acylcarnitine species, stratified by sex, CoLaus|PsyCoLaus study, Lausanne, Switzerland.

		**Weight (kg)**		**BMI (kg/m²)**		**Waist (cm)**		**Hip (cm)**		**WHR**	
		**Female**	**Male**	**Female**	**Male**	**Female**	**Male**	**Female**	**Male**	**Female**	**Male**
Short chain	Deoxycarnitine	0.025	0.055	0.003	0.000	-0.024	-0.020	-0.030	-0.021	-0.007	-0.009
	Acetylcarnitine	-0.092	-0.039	-0.082	-0.059	-0.063	-0.063	-0.061	-0.068	-0.031	-0.015
	Propionylcarnitine	**0.140**	**0.180**	**0.187**	**0.217**	**0.181**	**0.224**	**0.138**	**0.139**	**0.123**	**0.182**
	Butyrylcarnitine	0.038	0.067	0.062	0.056	0.070	0.063	0.050	0.032	0.050	0.058
	Hydroxybutyrylcarnitine	-0.037	-0.020	-0.027	-0.025	-0.002	-0.023	-0.007	-0.035	0.003	0.009
	Glutarylcarnitine	0.102	0.064	0.076	0.010	0.022	-0.012	0.036	-0.029	-0.010	0.014
	Hydroxyvalerylcarnitine	0.009	0.016	0.048	0.050	0.030	0.057	0.034	0.013	0.008	0.075
	Isovalerylcarnitine	**0.157**	**0.232**	**0.157**	**0.264**	**0.136**	**0.255**	**0.149**	**0.195**	0.037	**0.168**
	Tiglylcarnitine	-0.022	0.064	-0.009	0.070	-0.034	0.047	-0.016	0.019	-0.035	0.052
Medium-chain	Adipoylcarnitine	0.063	0.096	0.062	0.034	0.052	0.052	0.057	0.052	0.012	0.022
	Hexanoylcarnitine	0.066	0.075	0.018	0.036	0.029	0.065	0.043	0.039	-0.008	0.058
	Octanoylcarnitine	0.023	0.026	-0.027	-0.038	-0.013	-0.015	0.001	-0.008	-0.022	-0.011
	Octenoylcarnitine	**0.143**	0.113	**0.120**	0.126	**0.118**	0.105	**0.140**	0.077	0.020	0.070
	Decanoylcarnitine	0.023	-0.008	-0.031	-0.077	-0.012	-0.053	-0.001	-0.040	-0.018	-0.033
	Decenoylcarnitine	-0.028	-0.044	-0.070	-0.102	-0.024	-0.077	-0.026	-0.060	-0.010	-0.046
	Lauroylcarnitine	0.003	-0.027	-0.055	-0.096	-0.026	-0.083	-0.023	-0.060	-0.015	-0.056
	Hydroxydodecanoylcarnitine	-0.068	-0.046	**-0.113**	-0.105	-0.071	-0.095	-0.054	-0.064	-0.051	-0.069
	Dodecenoylcarnitine	0.021	0.044	-0.026	-0.012	0.008	0.005	0.011	0.008	-0.002	0.001
Long-chain	Myristolycarnitine	-0.013	-0.001	-0.080	-0.074	-0.042	-0.042	-0.034	-0.021	-0.028	-0.037
	Hydroxytetradecanoylcarnitine	-0.030	-0.023	-0.057	-0.008	-0.015	-0.025	-0.054	-0.100	0.042	0.078
	Tetradecenoylcarnitine	-0.031	-0.070	-0.074	-0.119	-0.046	-0.112	-0.035	-0.094	-0.033	-0.062
	Tetradecanedienoylcarnitine	-0.028	-0.025	-0.062	-0.073	-0.023	-0.074	-0.037	-0.062	0.006	-0.041
	Palmitoylcarnitine	0.010	0.037	-0.022	0.029	0.012	0.033	0.006	0.012	0.010	0.040
	Hexadecenoylcarnitine	-0.007	-0.051	-0.058	-0.096	-0.040	-0.077	-0.022	-0.061	-0.040	-0.045
	Heptadecanoylcarnitine	-0.044	0.019	-0.101	-0.042	-0.076	-0.027	-0.089	-0.031	-0.012	-0.006

	Stearoylcarnitine	-0.059	-0.050	**-0.114**	-0.086	-0.097	-0.101	-0.084	-0.106	-0.056	-0.034
	Oleoylcarnitine	-0.063	-0.037	-0.082	-0.045	-0.059	-0.017	-0.041	-0.036	-0.048	0.019
	Octadecadienoylcarnitine	-0.006	-0.020	-0.003	-0.063	0.019	-0.031	0.051	0.029	-0.031	-0.081
	Arachidonylcarnitine	0.000	-0.011	0.009	-0.004	0.043	-0.007	0.043	-0.008	0.014	0.000
	Free carnitine	**0.129**	**0.139**	**0.184**	**0.169**	**0.160**	**0.171**	**0.132**	0.104	0.094	**0.140**

BIA, bioimpedance analysis; BMI, body mass index; BRI, body roundness index; BSI, body shape index; WHR, waist to hip ratio; WHtR, waist to height ratio. Results are expressed as multivariable-adjusted beta coefficients. P-values were adjusted for multiple comparisons using the False Discovery Rate method according to Benjamini-Hochberg and statistically significant ($p<0.001$) coefficients are indicated in bold. Statistical analysis by linear regression adjusting on age (continuous), smoking status (never, former, current), presence of a diet (yes, no), and sedentary status (yes, no).

Supplementary table S7 (continued) Multivariable associations between anthropometric markers and acylcarnitine species, stratified by sex, CoLaus|PsyCoLaus study, Lausanne, Switzerland.

		**BIA (% fat mass)**		**Conicity index**		**WHtR**		**BRI**		**BSI**	
		**Female**	**Male**	**Female**	**Male**	**Female**	**Male**	**Female**	**Male**	**Female**	**Male**
Short chain	Deoxycarnitine	-0.028	-0.008	-0.054	-0.065	-0.038	-0.059	-0.037	-0.057	-0.059	-0.069
	Acetylcarnitine	0.002	-0.074	-0.018	-0.049	-0.052	-0.068	-0.051	-0.069	0.007	-0.031
	Propionylcarnitine	**0.132**	**0.162**	**0.130**	**0.161**	**0.194**	**0.219**	**0.190**	**0.219**	0.079	0.101
	Butyrylcarnitine	0.037	0.037	0.062	0.039	0.079	0.047	0.077	0.046	0.046	0.024
	Hydroxybutyrylcarnitine	0.039	-0.014	0.026	-0.009	0.004	-0.022	0.006	-0.024	0.036	0.000
	Glutarylcarnitine	0.032	0.028	-0.050	-0.062	0.000	-0.051	0.000	-0.051	-0.076	-0.071
	Hydroxyvalerylcarnitine	0.061	0.032	0.023	0.062	0.050	0.073	0.047	0.070	0.009	0.051
	Isovalerylcarnitine	0.081	0.111	0.064	**0.160**	**0.122**	**0.240**	**0.119**	**0.238**	0.018	0.084
	Tiglylcarnitine	-0.021	-0.020	-0.038	0.009	-0.025	0.041	-0.029	0.040	-0.038	-0.014
Medium-chain	Adipoylcarnitine	0.060	0.066	0.021	0.014	0.045	0.002	0.045	0.002	0.002	0.004
	Hexanoylcarnitine	0.012	0.057	0.007	0.050	0.000	0.035	-0.002	0.034	0.002	0.043
	Octanoylcarnitine	-0.017	0.043	-0.016	-0.018	-0.039	-0.052	-0.041	-0.053	-0.008	-0.007
	Octenoylcarnitine	0.089	0.092	0.061	0.044	0.094	0.094	0.091	0.094	0.028	0.005
	Decanoylcarnitine	-0.022	0.030	-0.012	-0.044	-0.041	-0.089	-0.043	-0.090	-0.002	-0.022
	Decenoylcarnitine	-0.020	0.013	0.006	-0.049	-0.046	-0.103	-0.044	-0.103	0.028	-0.018
	Lauroylcarnitine	-0.037	0.002	-0.015	-0.074	-0.057	-0.118	-0.058	-0.119	0.002	-0.047
	Hydroxydodecanoylcarnitine	-0.032	-0.036	-0.030	-0.078	-0.092	-0.121	-0.089	-0.122	0.004	-0.049
	Dodecenoylcarnitine	0.011	0.030	0.014	-0.011	-0.019	-0.032	-0.021	-0.033	0.023	-0.007
Long-chain	Myristolycarnitine	-0.038	-0.024	-0.021	-0.032	-0.077	-0.083	-0.076	-0.085	0.004	-0.010
	Hydroxytetradecanoylcarnitine	0.017	-0.004	0.016	-0.023	-0.030	-0.012	-0.031	-0.011	0.034	-0.021
	Tetradecenoylcarnitine	-0.024	-0.012	-0.023	-0.084	-0.067	-0.129	-0.067	-0.130	-0.001	-0.050
	Tetradecanedienoylcarnitine	-0.008	-0.021	0.005	-0.072	-0.040	-0.098	-0.040	-0.099	0.024	-0.053
	Palmitoylcarnitine	0.017	0.018	0.022	0.020	-0.008	0.024	-0.006	0.022	0.029	0.012
	Hexadecenoylcarnitine	-0.012	-0.014	-0.032	-0.049	-0.066	-0.094	-0.065	-0.095	-0.016	-0.019
	Heptadecanoylcarnitine	-0.077	-0.054	-0.051	-0.037	-0.103	-0.066	-0.104	-0.065	-0.023	-0.026

Stearoylcarnitine	-0.055	-0.040	-0.071	-0.099	**-0.123**	-0.115	**-0.121**	-0.114	-0.039	-0.079
Oleoylcarnitine	-0.019	-0.026	-0.029	0.016	-0.066	-0.016	-0.063	-0.017	-0.007	0.033
Octadecadienoylcarnitine	-0.019	-0.073	0.033	-0.011	0.020	-0.055	0.018	-0.057	0.035	0.009
Arachidonylcarnitine	-0.003	-0.008	0.061	-0.002	0.047	-0.001	0.046	-0.002	0.061	0.000
Free carnitine	**0.139**	**0.126**	0.101	0.121	**0.178**	**0.169**	**0.174**	**0.171**	0.048	0.074

BIA, bioimpedance analysis; BMI, body mass index; BRI, body roundness index; BSI, body shape index; WHR, waist to hip ratio; WHtR, waist to height ratio. Results are expressed as multivariable-adjusted beta coefficients. P-values were adjusted for multiple comparisons using the False Discovery Rate method according to Benjamini-Hochberg and statistically significant ($p<0.001$) coefficients are indicated in bold. Statistical analysis by linear regression adjusting on age (continuous), smoking status (never, former, current), presence of a diet (yes, no), and sedentary status (yes, no).

Supplementary table S7 (continued) Multivariable associations between anthropometric markers and acylcarnitine species, stratified by sex, CoLaus|PsyCoLaus study, Lausanne, Switzerland.

		Adiponectin (ng/mL)		Leptin (ng/mL)		Grip strength (kg)	
		Female	Male	Female	Male	Female	Male
Short chain	Deoxycarnitine	-0.017	-0.020	0.004	0.015	0.091	0.124
	Acetylcarnitine	-0.004	0.047	**-0.117**	0.023	-0.008	0.048
	Propionylcarnitine	-0.094	-0.094	**0.181**	0.113	-0.006	0.025
	Butyrylcarnitine	-0.018	-0.057	0.091	0.085	0.004	0.077
	Hydroxybutyrylcarnitine	-0.023	0.082	-0.095	0.079	-0.021	0.014
	Glutarylcarnitine	-0.019	-0.017	0.047	0.035	0.026	0.072
	Hydroxyvalerylcarnitine	-0.036	-0.056	0.049	0.040	0.042	0.086
	Isovalerylcarnitine	-0.090	-0.093	**0.142**	0.106	0.029	0.089
	Tiglylcarnitine	0.034	-0.044	0.020	0.066	0.038	0.121
Medium-chain	Adipoylcarnitine	0.007	0.066	0.026	0.112	-0.019	0.019
	Hexanoylcarnitine	0.023	-0.011	-0.001	0.064	0.057	0.105
	Octanoylcarnitine	0.031	-0.010	-0.027	0.030	0.057	0.084
	Octenoylcarnitine	-0.053	-0.060	0.057	0.034	0.022	0.025
	Decanoylcarnitine	0.026	0.005	-0.028	0.037	0.080	0.081
	Decenoylcarnitine	0.058	0.066	-0.063	0.079	0.077	0.057
	Lauroylcarnitine	0.034	0.037	-0.047	0.045	0.093	0.078
	Hydroxydodecanoylcarnitine	0.015	0.058	**-0.117**	0.040	0.051	0.053
	Dodecenoylcarnitine	0.013	0.002	-0.039	0.038	0.050	0.090
Long-chain	Myristolycarnitine	0.045	0.070	-0.069	0.055	0.073	0.074
	Hydroxytetradecanoylcarnitine	-0.008	0.010	-0.036	0.065	0.028	0.079
	Tetradecenoylcarnitine	0.052	0.057	-0.078	0.022	0.065	0.059
	Tetradecanedienoylcarnitine	0.033	0.055	-0.086	0.099	0.043	0.058
	Palmitoylcarnitine	0.023	0.020	-0.058	0.030	0.031	0.102
	Hexadecenoylcarnitine	0.073	0.113	-0.073	0.065	0.025	0.050
	Heptadecanoylcarnitine	**0.130**	0.103	-0.109	0.005	-0.003	0.053
	Stearoylcarnitine	0.051	0.063	**-0.129**	-0.044	0.004	0.023
	Oleoylcarnitine	0.003	0.020	-0.116	-0.056	-0.017	0.054
	Octadecadienoylcarnitine	0.017	0.065	-0.042	-0.032	0.032	0.048
	Arachidonylcarnitine	0.019	-0.003	-0.004	0.005	-0.003	0.066
	Free carnitine	-0.056	-0.105	**0.140**	0.062	0.010	0.082

BIA, bioimpedance analysis; BMI, body mass index; BRI, body roundness index; BSI, body shape index; WHR, waist to hip ratio; WHtR, waist to height ratio. Results are expressed as multivariable-adjusted beta coefficients. P-values were adjusted for multiple comparisons using the False Discovery Rate method according to Benjamini-Hochberg and statistically significant ($p<0.001$) coefficients are indicated in bold. Statistical analysis by linear regression adjusting on age (continuous), smoking status (never, former, current), presence of a diet (yes, no), and sedentary status (yes, no).

Supplementary table S8. Levels of acylcarnitine species between pre- and post-menopausal female participants, CoLaus|PsyCoLaus study, Lausanne, Switzerland.

		**Premenopaused N=509**	**Postmenopaused N=781**	**p-value**
Short chain	Deoxycarnitine	665.7 [573.6-767.5]	686.0 [589.6-787.1]	0.004
	Acetylcarnitine	6517 [5144-8061]	7498 [5985-9563]	<0.001
	Propionylcarnitine	294.9 [242.3-357.3]	344.0 [280.6-421.5]	<0.001
	Butyrylcarnitine	145.1 [111.5-184.0]	163.1 [128.3-214.1]	<0.001
	Hydroxybutyrylcarnitine	21.8 [14.8-33.0]	26.1 [18.4-39.0]	<0.001
	Glutarylcarnitine	44.7 [33.8-55.5]	44.3 [34.7-56.2]	0.58
	Hydroxyvalerylcarnitine	18.8 [14.9-23.4]	21.1 [16.7-25.7]	<0.001
	Isovalerylcarnitine	67.3 [53.0-83.2]	76.7 [61.6-93.9]	<0.001
	Tiglylcarnitine	9.3 [6.4-12.7]	11.0 [7.7-14.9]	<0.001
Medium-chain	Adipoylcarnitine	19.4 [14.1-29.1]	20.6 [15.3-27.8]	0.24
	Hexanoylcarnitine	29.4 [22.4-37.5]	35.8 [28.9-47.1]	<0.001
	Octanoylcarnitine	94.1 [67.2-127.5]	112.1 [82.4-153.1]	<0.001
	Octenoylcarnitine	53.3 [35.3-79.0]	64.7 [42.7-89.5]	<0.001
	Decanoylcarnitine	155.3 [111.2-220.1]	185.1 [137.0-253.7]	<0.001
	Decenoylcarnitine	60.0 [45.2-81.5]	76.6 [57.4-101.4]	<0.001
	Lauroylcarnitine	44.1 [32.9-62.4]	53.5 [40.0-74.2]	<0.001
	Hydroxydodecanoylcarnitine	8.6 [5.7-12.2]	11.3 [7.8-15.4]	<0.001
	Dodecenoylcarnitine	86.0 [64.1-108.1]	102.5 [80.2-131.9]	<0.001
Long-chain	Myristolycarnitine	20.9 [16.4-26.2]	25.2 [20.0-31.6]	<0.001
	Hydroxytetradecanoylcarnitine	3.6 [2.2-5.5]	4.9 [3.1-7.2]	<0.001
	Tetradecenoylcarnitine	54.8 [40.2-75.0]	68.8 [49.0-91.2]	<0.001
	Tetradecanedienoylcarnitine	36.5 [26.9-51.6]	45.0 [32.9-61.2]	<0.001
	Palmitoylcarnitine	102.9 [85.5-119.5]	114.5 [96.3-133.9]	<0.001
	Hexadecenoylcarnitine	25.0 [19.2-32.5]	31.6 [24.7-40.0]	<0.001
	Heptadecanoylcarnitine	3.5 [2.6-4.9]	4.4 [3.0-5.8]	<0.001
	Stearoylcarnitine	38.7 [30.7-48.3]	46.2 [36.7-57.7]	<0.001
	Oleoylcarnitine	156.9 [121.1-192.6]	177.3 [143.3-221.8]	<0.001
	Octadecadienoylcarnitine	41.7 [32.6-53.4]	46.2 [38.2-60.7]	<0.001
	Arachidonylcarnitine	3.2 [1.9-4.8]	4.3 [2.9-6.1]	<0.001
	Free carnitine	30.1 [25.9-34.2]	34.4 [29.9-39.1]	<0.001

Results are expressed as median [interquartile range]. Statistical analysis by Wilcoxon rank-sum test. All values in nMol except free carnitine (μMol).

Supplementary table S9. Multivariable associations between anthropometric markers and acylcarnitine species, after excluding early weight gainers (≥5 kg at first follow-up), CoLaus|PsyCoLaus study, Lausanne, Switzerland.

		Weight (kg)	BMI (kg/m^2)	Waist (cm)	Hip (cm)	Waist/hip ratio	BIA (% fat mass)	Conicity index	Waist to heigh ratio	Body roundness index	Body shape index	Adiponectin (ng/mL)	Leptin (ng/mL)	Grip strength (kg)
	Deoxycarnitine	0.034	-0.003	-0.026	-0.032	-0.010	-0.022	-0.065	-0.056	**-0.055**	-0.071	-0.019	0.004	0.080
	Acetylcarnitine	-0.051	-0.068	-0.054	-0.060	-0.020	-0.018	-0.026	-0.055	-0.054	-0.006	0.006	-0.070	0.017
	Propionylcarnitine	**0.136**	**0.196**	**0.186**	**0.144**	**0.131**	0.109	**0.143**	**0.206**	0.204	**0.091**	**-0.090**	**0.135**	**0.014**
	Butyrylcarnitine	0.041	0.057	0.059	0.043	0.045	0.029	0.050	0.065	0.063	0.036	-0.027	0.077	0.026
Short-chain	Hydroxybutyrylcarnitine	-0.021	-0.022	-0.008	-0.015	0.005	0.014	0.011	-0.005	-0.005	0.020	0.007	-0.038	0.000
	Glutarylcarnitine	0.071	0.049	0.008	0.015	-0.002	0.022	-0.054	-0.020	-0.021	-0.074	-0.019	0.037	0.033
	Hydroxyvalerylcarnitine	0.012	0.047	0.037	0.028	0.029	0.036	0.035	0.057	0.054	0.023	-0.045	0.039	0.046
	Isovalerylcarnitine	**0.169**	**0.209**	0.181	**0.178**	**0.089**	0.074	**0.107**	**0.179**	0.177	**0.048**	**-0.087**	**0.116**	**0.046**
	Tiglylcarnitine	0.019	0.023	0.000	0.003	-0.003	-0.012	-0.021	-0.001	**-0.003**	-0.030	-0.003	0.028	0.060
	Adipoylcarnitine	0.055	0.051	0.043	0.052	0.011	0.042	0.016	0.031	0.031	0.000	0.017	0.036	-0.005
	Hexanoylcarnitine	0.050	0.021	0.032	0.042	0.005	0.017	0.016	0.007	0.006	0.011	0.019	0.007	0.039
	Octanoylcarnitine	0.018	-0.026	-0.011	0.001	-0.017	-0.002	-0.016	-0.039	-0.041	-0.008	0.026	-0.017	0.034
	Octenoylcarnitine	**0.104**	**0.117**	0.099	0.116	**0.031**	0.065	**0.050**	0.090	0.087	**0.016**	-0.047	**0.044**	**0.015**
Medium-chain	Decanoylcarnitine	0.011	-0.038	-0.018	-0.008	-0.018	-0.007	-0.018	-0.049	-0.051	-0.006	0.024	-0.017	0.043
	Decenoylcarnitine	-0.027	-0.079	-0.039	-0.038	-0.019	-0.007	-0.011	-0.064	-0.063	0.014	0.057	-0.027	0.043
	Lauroylcarnitine	-0.005	-0.063	-0.038	-0.032	-0.023	-0.020	-0.030	-0.072	-0.073	-0.011	0.035	-0.029	0.051
	Hydroxydodecanoylcarnitine	**-0.046**	**-0.105**	-0.075	-0.060	**-0.050**	-0.028	-0.050	-0.104	-0.102	-0.020	0.030	-0.057	0.034
	Dodecenoylcarnitine	0.026	-0.020	0.006	0.011	-0.002	0.013	0.005	-0.024	-0.025	0.012	0.012	-0.021	0.044
	Myristolycarnitine	-0.006	-0.076	-0.038	-0.030	-0.027	-0.026	-0.024	-0.078	-0.078	-0.001	0.048	-0.039	0.044
	Hydroxytetradecanoylcarnitine	-0.019	-0.033	-0.016	-0.071	0.051	0.005	-0.001	-0.021	-0.021	0.010	-0.012	-0.007	0.038
	Tetradecenoylcarnitine	**-0.036**	-0.086	-0.062	-0.054	-0.037	-0.016	-0.041	-0.086	-0.086	-0.016	0.050	-0.049	0.040
	Tetradecanedienoylcarnitine	-0.019	-0.063	-0.038	-0.044	-0.012	-0.010	-0.023	-0.061	-0.062	-0.005	0.038	-0.034	0.035
Long-chain	Palmitoylcarnitine	0.019	-0.002	0.020	0.008	0.023	0.011	0.023	0.006	0.006	0.025	0.018	-0.030	0.047
	Hexadecenoylcarnitine	-0.018	-0.066	-0.045	-0.034	-0.033	-0.009	-0.034	-0.072	-0.071	-0.015	0.079	-0.040	0.024
	Heptadecanoylcarnitine	-0.013	**-0.076**	-0.050	-0.065	**-0.010**	**-0.051**	-0.044	-0.088	-0.088	-0.023	0.101	-0.068	0.020
	Stearoylcarnitine	**-0.041**	**-0.098**	-0.087	-0.090	**-0.039**	-0.035	-0.078	-0.117	-0.116	-0.053	**0.051**	**-0.091**	0.013
	Oleoylcarnitine	-0.037	-0.058	-0.033	-0.037	-0.012	-0.014	-0.008	-0.040	-0.039	0.009	0.006	-0.084	0.015

Octadecadienoylcarnitine	-0.009	-0.027	-0.002	0.037	-0.042	-0.032	0.014	-0.011	-0.013	0.023	0.029	-0.034	0.026
Arachidonylcarnitine	0.000	0.011	0.025	0.024	0.013	-0.001	0.038	0.034	0.034	0.037	0.001	-0.001	0.033
Free carnitine	**0.111**	**0.171**	0.152	**0.125**	**0.100**	0.102	**0.112**	**0.175**	0.173	**0.065**	**-0.069**	**0.098**	**0.038**

BIA, bioimpedance analysis; BMI, body mass index. Results are expressed as multivariable-adjusted beta coefficients. P-values are adjusted for multiple comparisons using the False Discovery Rate method of Benjamini-Hochberg and statistically significant coefficients ($p<0.001$) are indicated in bold. Statistical analysis by linear regression adjusting on sex (male, female), age (continuous), smoking status (never, former, current), presence of a diet (yes, no), and sedentary status (yes, no).

Supplementary table S10. Multivariable-adjusted differences in acylcarnitine concentrations associated with ≥5 kg weight loss or ≥5 kg weight gain compared with stable weight, CoLaus|PsyCoLaus study, Lausanne, Switzerland.

		Weight changes over 5 years		Weight changes over 10 years	
		Lost 5+kg	Gained 5+ kg	Lost 5+kg	Gained 5+ kg
	Deoxycarnitine	-8.87 (-44.3 ; 26.57)	.0369 (-22.1 ; 22.17)	-21.2 (-52.7 ; 10.37)	4.094 (-16.9 ; 25.10)
	Acetylcarnitine	**-700. (-1271 ; -130.)**	-95.2 (-451 ; 261.1)	49.11 (-462. ; 560.7)	271.9 (-68.4 ; 612.3)
	Propionylcarnitine	-14.2 (-40.1 ; 11.72)	6.171 (-10 ; 22.36)	7.816 (-15.7 ; 31.34)	3.269 (-12.3 ; 18.92)
	Butyrylcarnitine	**-23.8 (-45.0 ; -2.62)**	-1.11 (-14.3 ; 12.12)	-9.40 (-28.9 ; 10.19)	4.987 (-8.04 ; 18.02)
Short-chain	Hydroxybutyrylcarnitine	**-5.67 (-11.0 ; -.325)**	1.197 (-2.14 ; 4.538)	.8060 (-4.04 ; 5.659)	**4.135 (.9067 ; 7.364)**
	Glutarylcarnitine	-.701 (-4.93 ; 3.534)	1.136 (-1.50 ; 3.782)	-1.52 (-5.35 ; 2.306)	-.134 (-2.68 ; 2.415)
	Hydroxyvalerylcarnitine	-1.50 (-3.43 ; .4316)	**1.957 (.7507 ; 3.164)**	-.297 (-2.00 ; 1.414)	**1.792 (.6536 ; 2.931)**
	Isovalerylcarnitine	-4.46 (-12.1 ; 3.197)	2.924 (-1.86 ; 7.710)	-.354 (-7.32 ; 6.613)	4.099 (-.536 ; 8.734)
	Tiglylcarnitine	-.773 (-2.06 ; .5180)	.3573 (-.450 ; 1.165)	-.308 (-1.48 ; .8675)	.2194 (-.564 ; 1.003)
	Adipoylcarnitine	1.204 (-1.72 ; 4.133)	1.033 (-.796 ; 2.863)	1.810 (-.811 ; 4.433)	.5896 (-1.15 ; 2.334)
	Hexanoylcarnitine	-3.00 (-10.8 ; 4.836)	-1.46 (-6.36 ; 3.435)	.7675 (-6.67 ; 8.212)	**5.277 (.3240 ; 10.23)**
	Octanoylcarnitine	-14.5 (-49.9 ; 20.82)	-11.8 (-33.9 ; 10.25)	-3.95 (-37.6 ; 29.73)	16.30 (-6.10 ; 38.72)
	Octenoylcarnitine	-4.68 (-14.7 ; 5.407)	**6.486 (.1812 ; 12.79**)	-3.31 (-12.2 ; 5.662)	**9.331 (3.351 ; 15.31)**
Medium-chain	Decanoylcarnitine	-23.6 (-74.1 ; 26.80)	-19.5 (-51.0 ; 11.99)	-7.36 (-55.2 ; 40.49)	20.46 (-11.3 ; 52.30)
	Decenoylcarnitine	-2.07 (-10.1 ; 6.001)	-3.20 (-8.24 ; 1.837)	2.578 (-4.77 ; 9.929)	2.791 (-2.09 ; 7.682)
	Lauroylcarnitine	-5.58 (-15.5 ; 4.353)	-3.72 (-9.93 ; 2.479)	-.213 (-9.50 ; 9.082)	4.398 (-1.78 ; 10.58)
	Hydroxydodecanoylcarnitine	-.878 (-2.29 ; .5386)	-.758 (-1.64 ; .1266)	.5251 (-.744 ; 1.794)	.0603 (-.784 ; .9051)
	Dodecenoylcarnitine	-7.34 (-18.5 ; 3.846)	-5.21 (-12.2 ; 1.778)	-1.42 (-11.6 ; 8.830)	2.562 (-4.25 ; 9.384)
	Myristolycarnitine	-1.01 (-3.53 ; 1.514)	-.659 (-2.23 ; .9182)	1.277 (-1.03 ; 3.588)	1.244 (-.292 ; 2.782)
	Hydroxytetradecanoylcarnitine	-.011 (-.853 ; .8312)	-.065 (-.592 ; .4603)	.6451 (-.096 ; 1.387)	.2729 (-.220 ; .7666)
	Tetradecenoylcarnitine	-6.07 (-15.0 ; 2.911)	-3.62 (-9.23 ; 1.987)	.7571 (-7.45 ; 8.968)	4.304 (-1.15 ; 9.767)
Long-chain	Tetradecanedienoylcarnitine	-5.39 (-11.1 ; .3269)	-1.33 (-4.90 ; 2.239)	-.703 (-5.90 ; 4.501)	1.930 (-1.53 ; 5.393)
	Palmitoylcarnitine	-.753 (-6.89 ; 5.385)	.4322 (-3.40 ; 4.267)	**5.731 (.2541 ; 11.20)**	3.443 (-.200 ; 7.087)
	Hexadecenoylcarnitine	-.747 (-3.54 ; 2.052)	-.422 (-2.17 ; 1.326)	1.869 (-.688 ; 4.426)	1.569 (-.131 ; 3.271)
	Heptadecanoylcarnitine	.0463 (-.419 ; .5124)	-.205 (-.496 ; .0846)	.2119 (-.203 ; .6277)	-.107 (-.385 ; .1697)
	Stearoylcarnitine	-.964 (-4.42 ; 2.495)	-.179 (-2.34 ; 1.982)	1.863 (-1.26 ; 4.993)	.8274 (-1.25 ; 2.909)

Oleoylcarnitine	-1.39 (-15.2 ; 12.45)	-.611 (-9.26 ; 8.042)	8.341 (-4.13 ; 20.81)	7.165 (-1.13 ; 15.46)
Octadecadienoylcarnitine	-1.84 (-9.41 ; 5.719)	-1.24 (-5.97 ; 3.478)	**9.736 (2.817 ; 16.65)**	4.514 (-.088 ; 9.118)
Arachidonylcarnitine	-.073 (-.686 ; .5391)	.1599 (-.220 ; .5398)	.1107 (-.436 ; .6582)	.3062 (-.059 ; .6715)
Free carnitine	-.267 (-1.82 ; 1.290)	.4869 (-.486 ; 1.460)	.0910 (-1.30 ; 1.490)	.3450 (-.586 ; 1.276)

Values are presented as β coefficients with 95% confidence intervals and p-values from multivariable linear regression models. Weight-stable participants were used as the reference group. β coefficients represent the adjusted mean difference in circulating acylcarnitine concentrations among participants with ≥5 kg weight loss or ≥5 kg weight gain compared with those with stable weight. Models were adjusted for age (continuous), smoking status (never, former, current), presence of a diet (yes, no), and sedentary status (yes, no). Statistically significant associations ($p < 0.05$) are indicated in bold.
