# Supplementary material for: Association of Acylcarnitine Species and Anthropometry Markers in a Population-Based Apparently Healthy Cohort
Source: Metabolites. 2026 May 6;16(5):315. doi: 10.3390/metabo16050315 (PMC13208298; doi:10.3390/metabo16050315)
Supplement: Supplementary file 1 [file metabolites-16-00315-s001.zip › SupFigures_20260404.pptx]

## Slide 1
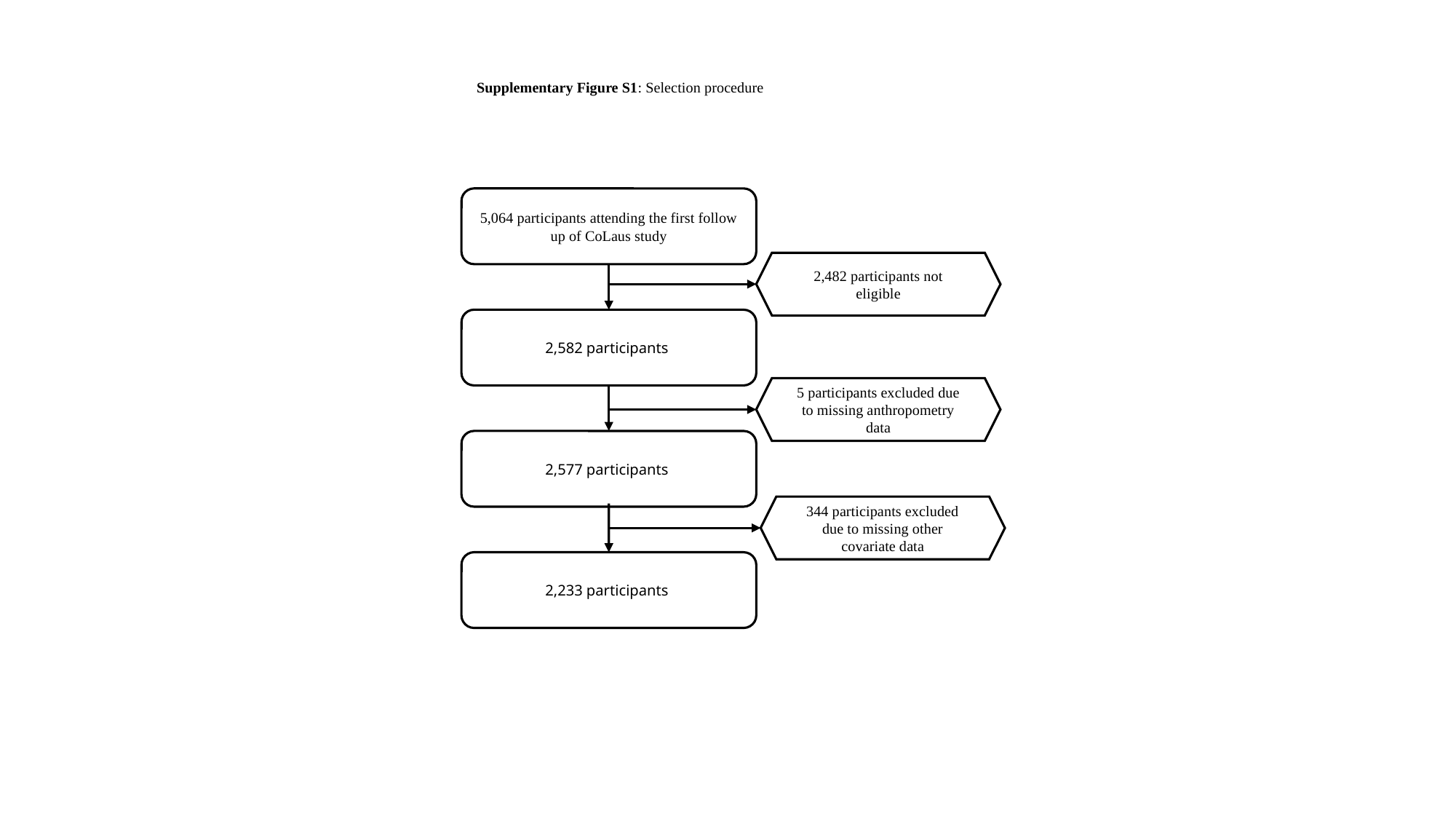

Supplementary Figure S1: Selection procedure
5,064 participants attending the first follow up of CoLaus study
2,482 participants not eligible
2,582 participants
5 participants excluded due to missing anthropometry data
2,577 participants
344 participants excluded due to missing other covariate data
2,233 participants

## Slide 2
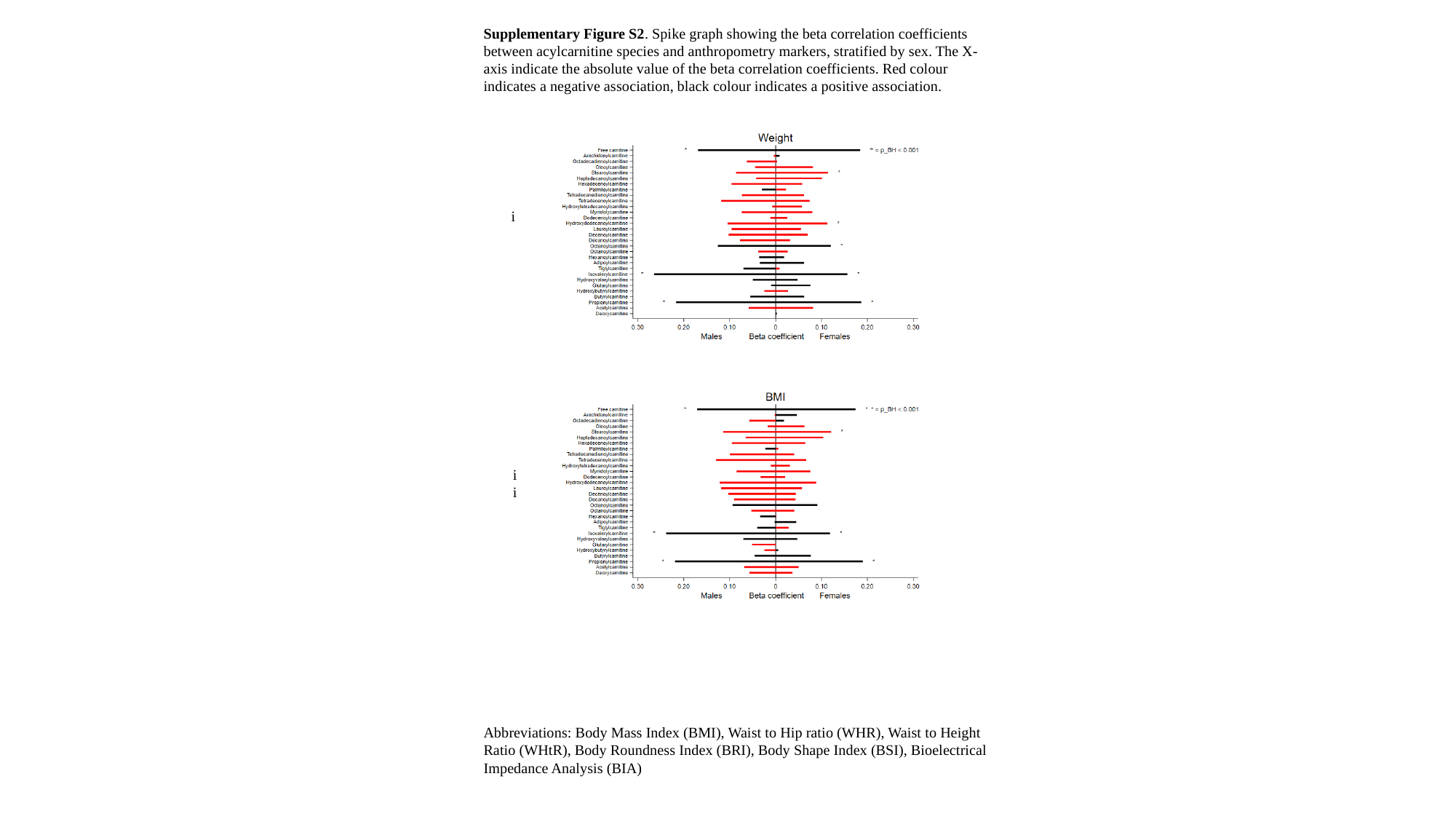

Supplementary Figure S2. Spike graph showing the beta correlation coefficients between acylcarnitine species and anthropometry markers, stratified by sex. The X-axis indicate the absolute value of the beta correlation coefficients. Red colour indicates a negative association, black colour indicates a positive association.
i
ii
Abbreviations: Body Mass Index (BMI), Waist to Hip ratio (WHR), Waist to Height Ratio (WHtR), Body Roundness Index (BRI), Body Shape Index (BSI), Bioelectrical Impedance Analysis (BIA)

## Slide 3
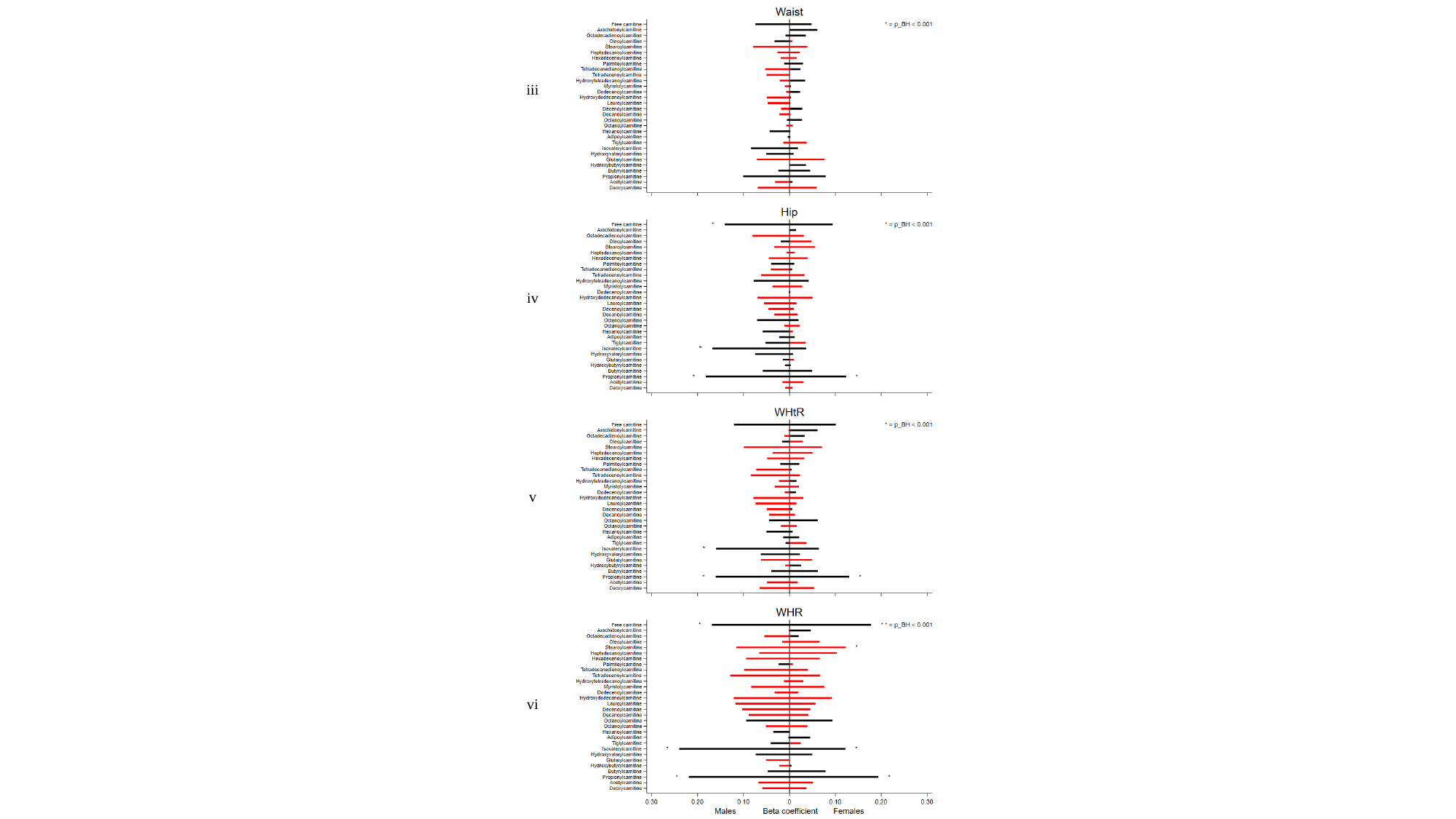

iii
iv
v
vi

## Slide 4
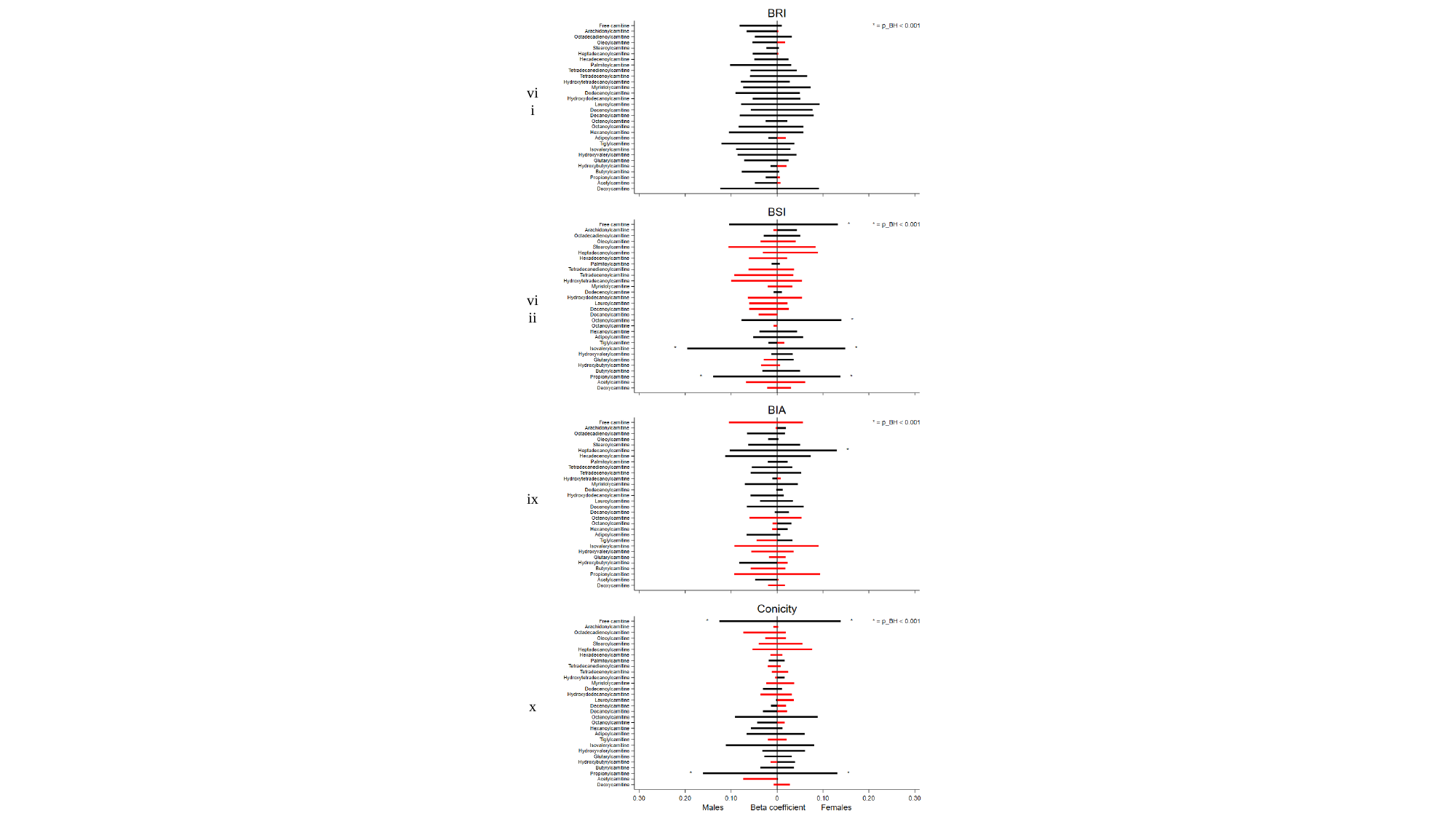

vii
viii
ix
x

## Slide 5
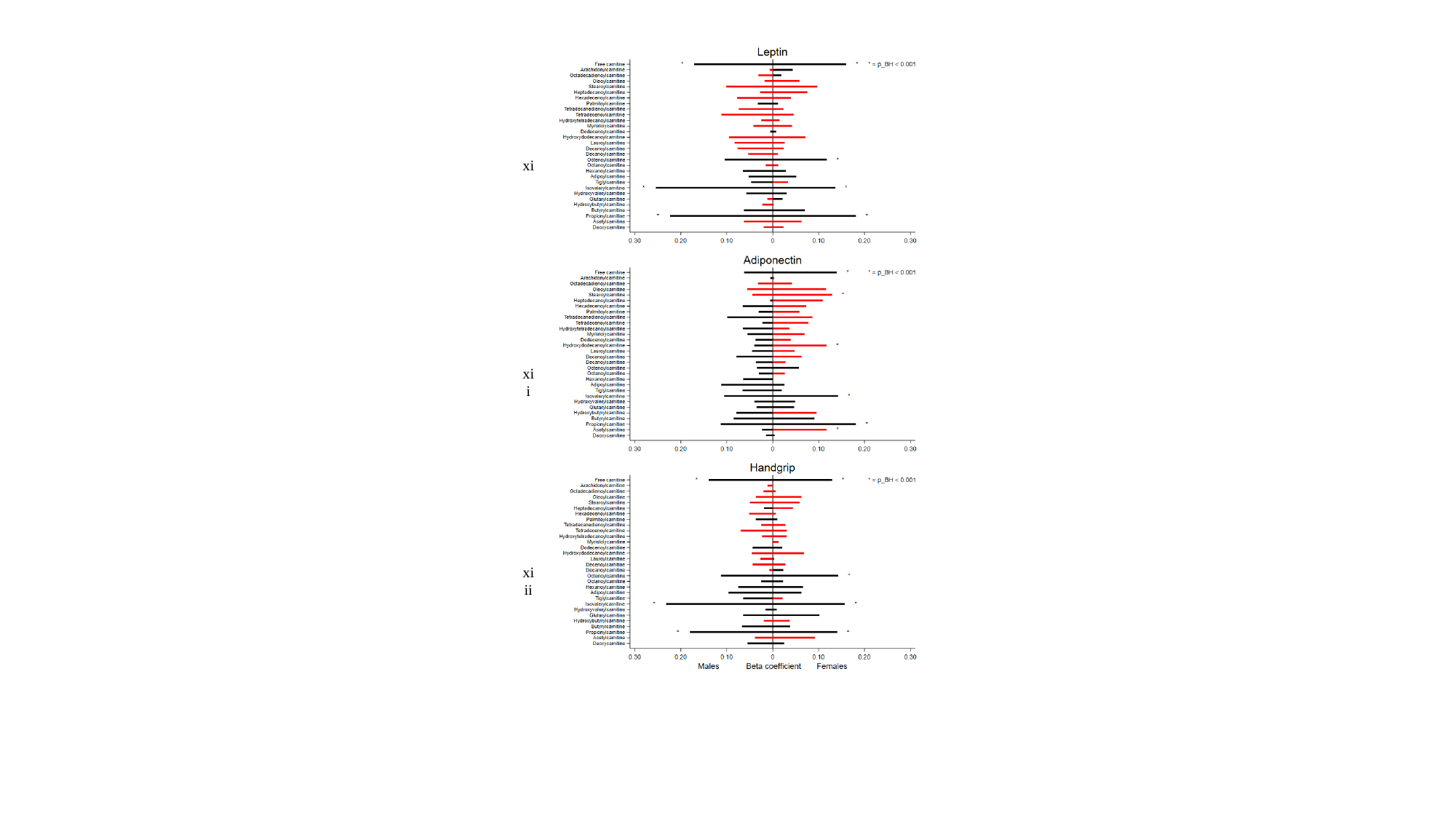

xi
xii
xiii

## Slide 6
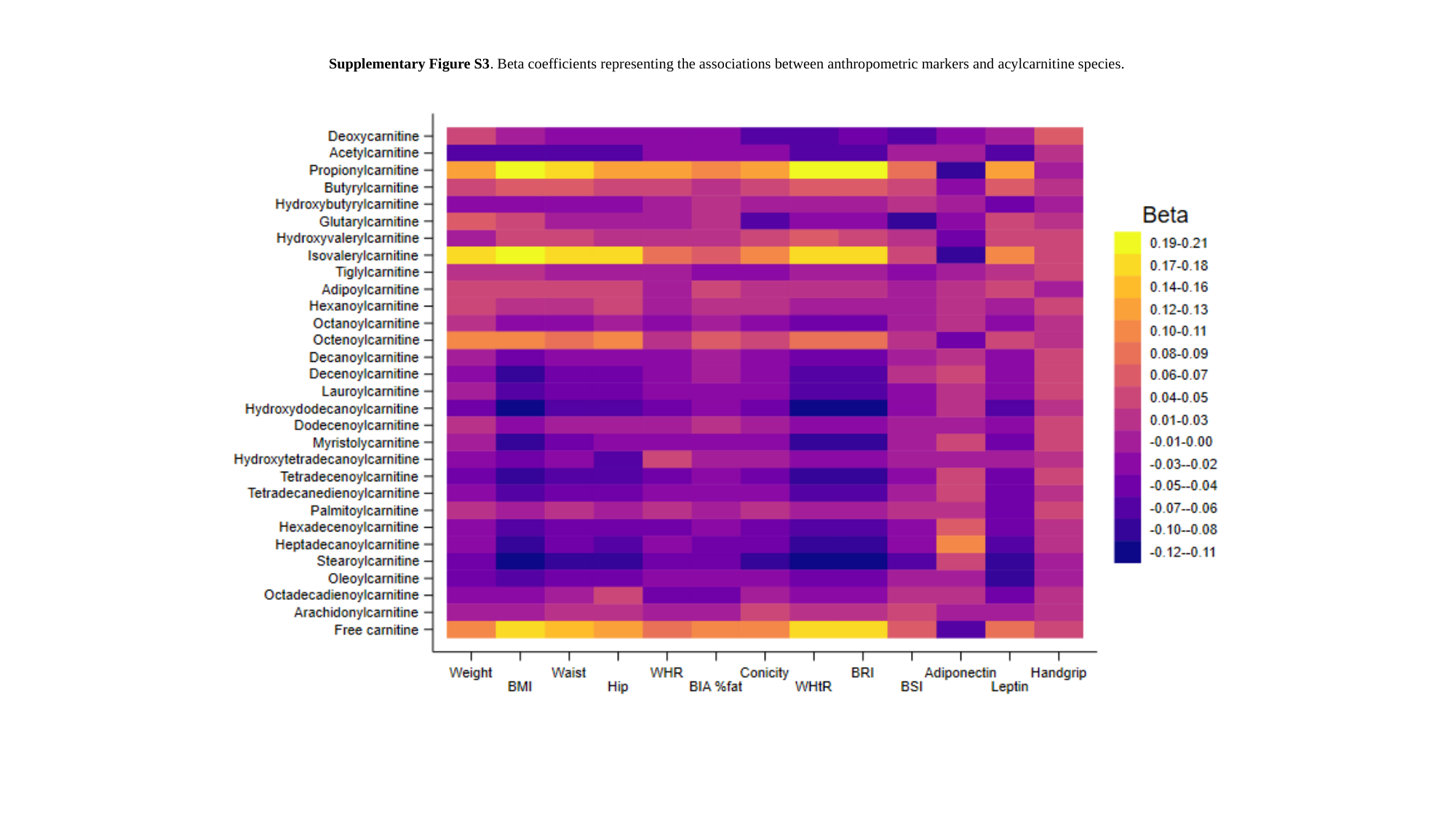

Supplementary Figure S3. Beta coefficients representing the associations between anthropometric markers and acylcarnitine species.
